# HyAdamC: A New Adam-Based Hybrid Optimization Algorithm for Convolution Neural Networks

**DOI:** 10.3390/s21124054

**Published:** 2021-06-12

**Authors:** Kyung-Soo Kim, Yong-Suk Choi

**Affiliations:** 1 Center for Computational Social Science, Hanyang University, Seoul 04763, Korea; kyungskim@hanyang.ac.kr; 2 Department of Computer Science and Engineering, Hanyang University, Seoul 04763, Korea

**Keywords:** deep learning, optimization, first-order optimization, gradient descent, adam optimization, convolution neural networks, image classification

## Abstract

As the performance of devices that conduct large-scale computations has been rapidly improved, various deep learning models have been successfully utilized in various applications. Particularly, convolution neural networks (CNN) have shown remarkable performance in image processing tasks such as image classification and segmentation. Accordingly, more stable and robust optimization methods are required to effectively train them. However, the traditional optimizers used in deep learning still have unsatisfactory training performance for the models with many layers and weights. Accordingly, in this paper, we propose a new Adam-based hybrid optimization method called HyAdamC for training CNNs effectively. HyAdamC uses three new velocity control functions to adjust its search strength carefully in term of initial, short, and long-term velocities. Moreover, HyAdamC utilizes an adaptive coefficient computation method to prevent that a search direction determined by the first momentum is distorted by any outlier gradients. Then, these are combined into one hybrid method. In our experiments, HyAdamC showed not only notable test accuracies but also significantly stable and robust optimization abilities when training various CNN models. Furthermore, we also found that HyAdamC could be applied into not only image classification and image segmentation tasks.

## 1. Introduction

As the computational power of graphic processing units (GPU) have further enhanced, various neural network models performing a large-scale computations have been actively studied. In particular, modern neural network models are consisted of deeper layers and more weights than traditional ones to maximize their performance. Accordingly, the latest deep learning models have shown notable abilities in many real-world applications, for example, computer visions (CV) [1,2], data analysis [3,4], personalized services [5,6], internet of things (IoT) [7,8], and natural language processing (NLP) [9,10], et al. Among them, particularly, the CV task involving image classification and image semantic segmentation is one of the applications in which the deep learning models have been most actively used.

Accordingly, many studies to improve the image processing ability of CNNs are being actively conducted. In particular, the recent many researches have focused on further enhancing their architectures by stacking a lot of layers sequentially. For example, GoogleNet [11], VGG [12], ResNet [13], DenseNet [14], and MobileNet [15] have shown successful image processing performance, especially, high-level image classification ability, in various application areas. It indicates that constructing the architecture of the CNNs using deeper layers and more weights can contribute to boost their image processing power.

Nevertheless, such large-scale architecture has two weaknesses. First, if a CNN model has a number of layers, the gradient becomes vanished gradually as it has been passed from the output to the input layers. Accordingly, the weights around the initial layers cannot be sufficiently trained, which is called “vanishing gradient problem”. Second, because they contain a great number of weight values, their loss function often has further complicated solution space (i.e., optimization terrain). Such complex terrain involves many saddle point or local minimums which makes searching its optimal weights extremely hard. Thus, it is important to not only construct the architectures with many layers and weights but also utilize robust and stable optimization methods that can boost the image processing performance of the CNN models.

Meanwhile, the traditional first-order optimization methods such as gradient descent (GD), stochastic GD (SGD) [16], SGD with momentum (SGDM) [17], and Adam [18] have been widely utilized to train the CNNs. Among them, the Adam optimizer has shown more improved solution search ability than the existing methods by utilizing the momentum and bias-correction methods. Accordingly, recent many studies have focused on improving the performance of Adam optimizer further or combining it with other optimization methods [19]. As the most representative cases, Adagrad [20], Yogi [21], Fromage [22], diffGrad [23], and TAdam [24] have successfully been proposed recently. However, they still have several defects in training latest CNNs. First, they suffer from determining an optimal search velocity at each training step, which often causes the overfitting problem or worsens their training and test accuracies [25,26]. Second, the existing momentum used in Adam optimizer can easily be skewed toward an inaccurate search direction made by outlier gradients [24]. Third, they often fail to find the approximate optimal weights because they have difficulty identifying the current state of the optimization terrain in the solution space spanned by the weights. Accordingly, the CNNs trained by the existing optimization methods often have unsatisfactory performance such as inaccurate image classification ability even though they could achieve better results.

To overcome such weaknesses of the existing optimization methods, we propose a hybrid and adaptive optimization method that utilizes various terrain information when searching the complicated solution space of CNNs. In this paper, we define our novel hybrid and adaptive first-order optimization method as “HyAdamC ”. The core strategy of HyAdamC is to accurately identify a current state of the optimization terrain and adaptively control its search velocity depending on the identified states. For this, HyAdamC analyzes the past and current gradients in terms of the initial, short, and long-term convergences and utilizes them to control the search velocity. In addition, HyAdamC further improves its search ability by preventing that the first momentum is distorted by any outlier gradients. Furthermore, HyAdamC effectively alleviates many problems from which the existing optimization methods have suffered by taking a hybrid approach that combines such various strategies with one. Such hybrid method makes more stable and robust solution search than the traditional optimization methods. The core characteristics of our HyAdamC are summarized as follows.

- First, we show how HyAdamC identifies a current state of the optimization terrain from the past momentum and gradients.
- Second, we propose three new velocity control functions that can adjust the search velocity elaborately depending on the current optimization states.
- Third, we propose a hybrid mechanism by concretely implementing how these are combined. Furthermore, we show how our hybrid method contributes to enhancing its optimization performance in training the CNNs.
- Fourth, we propose how the three velocity functions and the strategy to prevent outlier gradient are combined into one method. Accordingly, we show that such elastic hybrid method can significantly contribute to overcome the problems from which the existing optimization methods have suffered.

Accordingly, we can summary the contributions of this study as follows.

- First, we discovered a variety of useful information for searching an optimal weight in the solution space with any complicated terrains.
- Second, we showed that such various terrain information could be simply utilized by applying them to scale the search direction.
- Third, we concretely found that minimizing the unexpected affections caused by any outlier gradients could contribute significantly to determining its promising search direction by implementing the adaptive coefficient computation methods.
- Fourth, we showed that they could be combined as a hybrid optimization method with a detailed implementation.
- Fifth, we confirmed that our HyAdamC was theoretically valid by proving its upper regret bound mathematically.
- Sixth, we validated the practical optimization ability of HyAdamC by conducting comprehensive experiments with various CNN models for the image classification and image segmentation tasks.

Our article consists of seven sections as follows. In Section 2, we explain theoretical backgrounds required to understand the optimization methods used to train CNNs. In Section 3, we introduce the Adam optimizer and the latest first-order optimization methods briefly. In Section 4, we explain our new optimization method, i.e., HyAdamC, with detailed implementations. In Section 5, we show all the experiments performed to evaluate the optimization performance of HyAdamC and their results in detail. In Section 6, we discuss the experimental results in terms of each of the CNN models. Finally, we conclude this article and introduce our future study plan briefly in Section 7.

## 2. Preliminaries

In general, a neural network model, involving the CNNs, is formulated as

(1) M={N,W,f(x;W)}

where N is a set of neurons and W is a set of weights between any two neurons, and f(x;W) is an output function of the computational graph constituted by N and W for any input X. Let (x,y) be a data pair involved in a dataset and its predicted output be $y^{*}$. Then, a loss function to measure an error between the ground truth (GT) output of the input data and its predicted output (i.e., y and $y^{*}$) is formulated as

(2) $$L\left(y^{*},y\right)=\left\{l|y^{*}=f\left(x;w\right)\wedge l=loss\left(y,y^{*}\right)\right\}$$

where $loss(y,y^{*})$ is a loss value measured by any error functions such as the cross-entropy. Our goal is to find an approximate optimal weight $w^{*}$ which minimizes the loss function. Hence, the loss function L(x,y) is used as an objective function to evaluate a fitness of the weight. Accordingly, training a neural network is formulated as a typical search problem as

(3) $$w^{*}={argmin}_{w}\frac{\partial }{\partial w}L\left(f\left(x;w\right),y\right).$$

Meanwhile, the optimization algorithms for training neural networks are mainly divided into two approaches according to their fundamental solution search methods, i.e., the first-order and the second-order optimization methods, respectively [27,28]. The first-order optimization methods search an optimal weight by iteratively moving the weights found at each step to a direction of its gradient in the solution space. In particular, we need to notice that this update method is derived from the first-order Taylor series of the loss function, which is given by

(4) $$L\approx L\left(f\left(x;w_{0}\right),y\right)+\nabla_{w}L\left(f\left(x;w\right),y\right)$$

where $\nabla_{w}L\left(f\left(x;w\right),y\right)$ denotes a gradient of the loss function L(f(x;w),y) at each step, which is computed by

(5) $$\nabla_{w}L\left(f\left(x;w\right),y\right)={\left[\frac{\partial L}{\partial w_{1}},\frac{\partial L}{\partial w_{2}},\cdots ,\frac{\partial L}{\partial w_{n}}\right]}^{T}.$$

Then, the current weight $w_{t}$ is updated to $w_{t+1}$ by moving $w_{t}$ toward a direction of it gradient. For example, GD updates its current weight $w_{t}$ as

(6) $$w_{t+1}=w_{t}-\alpha \nabla_{w}L\left(f\left(x;w_{t}\right),y\right)$$

where α is a learning rate to control a strength of convergence.

On the other hands, the second-order optimization methods use a Hessian matrix of the loss function additionally to find the optimal weight [29]. Similar to the first-order method, the second-order optimization methods are also derived from the second-order Taylor series of the loss function, which is given by

(7) $$L\approx L\left(f\left(x;w_{0}\right),y\right)+\nabla_{w}L\left(f\left(x;w\right),y\right)+\frac{1}{2!}\nabla_{w}^{2}L\left(f\left(x;w\right),y\right)$$

where $\nabla_{w}^{2}L\left(f\left(x;w\right),y\right)$ is the Hessian matrix that is computed by taking a second differential of the loss function with respect to each parameter as

(8) $$\nabla_{w}^{2}L\left(f\left(x;w\right),y\right)=\left(\begin{array}{ccc} \frac{\partial^{2}L}{\partial^{2}w_{1}} & \cdots & \frac{\partial^{2}L}{\partial w_{1}\partial w_{n}}\\ \vdots & \ddots & \vdots \\ \frac{\partial^{2}L}{\partial w_{n}\partial w_{1}} & \cdots & \frac{\partial^{2}L}{\partial^{2}w_{n}} \end{array}\right).$$

The second-order methods determine its next search direction by combining the gradient with the Hessian matrix. For example, Newton’s methods, one of the second-order optimization methods [30], updates the current parameter $w_{t}$ as

(9) $$w_{t+1}=w_{t}-\alpha {\left[{\nabla_{w}}^{2}L\left(f\left(x;w_{t}\right),y\right)\right]}^{-1}\nabla_{w}L\left(f\left(x;w_{t}\right),y\right).$$

From Equations (6) and (9), we can expect that the second-order methods may have better optimization ability than the first-order ones. Nevertheless, these are not often used to train the neural networks because they need at least O($n^{2}$) space complexity to compute the *n* by *n* Hessian matrix [19]. Although many studies to alleviate these excessive computational overhead have been carried out, it is still a challenging issue to utilize these for training the neural networks when compared against the first-order methods. Hence, recent many studies are focusing on developing more effective first-order optimization methods.

## 3. Related Work

### 3.1. CNNs

**Figure 1:**
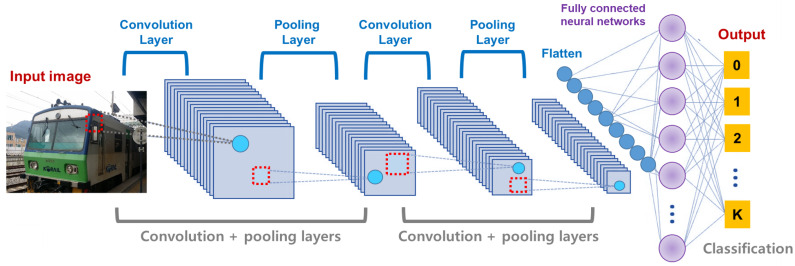
The overall architecture of CNNs.

The CNN is a neural network model proposed to train the data with region features. When the model was initially proposed, it did not receive much attention because of its excessive computational costs. However, as the performance of the computing device has drastically enhanced, it has recently became one of the most popular deep learning models. In particular, it has been successfully utilized in various applications such as the image classification, image semantic segmentation, and objective detection tasks [31,32,33,34,35]. Before discussing an optimization method to effective train the CNN, we need to understand its basic architecture and characteristics briefly. Different to the traditional neural networks, the CNN is composed of three layers, i.e., a convolution layer, a pooling layer, and a fully-connected layer. Figure 1 shows an overall architecture of the CNN. In detail, the convolution layer extracts region features from the input image using convolution operations and receptive fields. At this time, each feature is computed by a linear combination of the input values recognized within the receptive field. Then, the region feature is passed to the pooling layer. The pooling layer reduces a size of the region feature into smaller-dimensional one. For this, various pooling operations such as max pooling, average pooling, and min pooling are used. Finally, the reduced region feature is flatten and passed into an input of the fully connected neural network where it is classified into one class.

**Figure 2:**
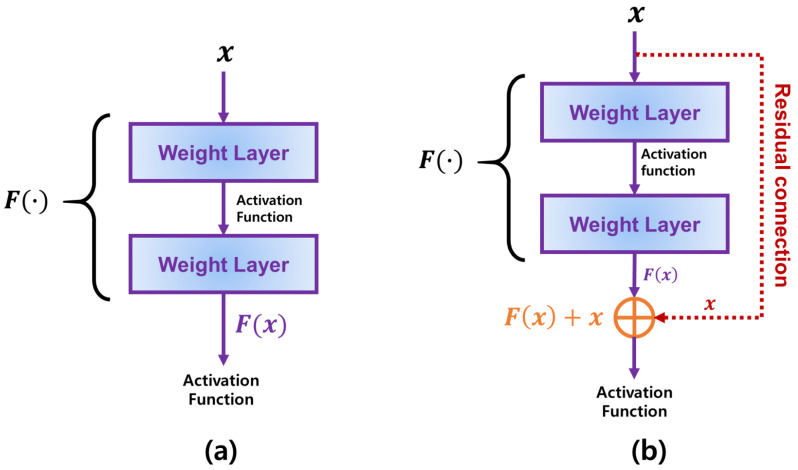
The comparison between the general connections and the residual connections used in ResNet. (**a**) describes how the input data x is passed into the weight layers in general CNN models with no residual connections. (**b**) shows how x is passed to both the weight layers and the residual connections in ResNet.

The architecture shown in Figure 1 implies that the CNN is especially effective to address a two-dimensional data such as an image. Different to the data that can be represented by a linear feature vector, an image should be analyzed considering a position information of each pixel in two-dimensional space. As the input image shown in Figure 1, a color data in each pixel is strongly affected by other pixels around it. Accordingly, many CNN models to effectively address complex image data have been actively proposed. For example, Karen Simonyan and Andrew Zisserman showed that constructing many layers in the CNNs could achieve better performance than the traditional CNNs by implementing CNNs with 16 or more layers, called VGG [12]. However, the CNN models involving many layers suffered from the vanishing gradient, which worsens an accuracy of the image classification. To overcome such weakness of the existing CNN models, ResNet utilizes additional weights called residual connections [13]. Figure 2 describes two conceptual diagrams of the general connection used in the existing CNNs and the residual connection utilized in ResNet, respectively. In the existing CNN models described in Figure 2a, a connection between any two layers is connected from the previous and current layers sequentially. In this case, if the number of layers drastically increases, the gradient becomes more and more vanished as it has been passed from the output to the input layers. On the other hands, Figure 2b shows the residual connection in ResNet where each block is connected by not only its parent one but also ascendant one directly simultaneously. Thus, ResNet can effectively alleviate the vanishing gradient problem when compared to the existing CNNs with no residual connections. Furthermore, Gao Huang, Zhuang Liu, and et al. proposed a further enhanced CNN model with the residual connections between all blocks, which is called DenseNet [14]. While the residual connections of ResNet is added into the general connection, DenseNet concatenates them into one connection and passes it into all following layers. Accordingly, DenseNet has more weights than those of ResNet.

That is, the modern CNN models such as ResNet and DenseNet could construct more layers than the traditional ones by introducing various auxiliary connections such the residual connections into their models. Tuhs, it is required to not only modify their architecture but also utilize a robust and stable optimization method in order to improve the image processing performance of such complicated CNNs, for example, an accurate image classification ability.

### 3.2. Overview of Optimization Methods for Machine Learning

As shown in Section 2, the first-order optimization methods determine its next search direction by referring the gradient of the loss function on the current weights. For this, the first-order methods compute its gradient using the first-order differential as explained in Equation (5). Many optimization methods to train not only the neural networks but also diverse machine learning models have been designed based on the first-order methods. For example, GD [19], SGD [16], RMSProp [36], and Adam [18] are typical first-order methods. Furthermore, Yogi [21], Fromage [22], diffGrad [23], and TAdam [24] also were designed based on the first-order methods.

On the other hands, the second-order method determines its next search direction from Hessian matrix of the loss function as shown in Equation (9). Accordingly, the second-order method can perform better solution search in the complicated optimization terrains with a lot of saddle points or local minimums [29]. Nevertheless, because the second-order methods need more computations than those of the first-order methods, it is a hard task to apply them directly to train the deep neural networks. To overcome such incredible computational complexity, recent many studies have focused on reducing its computations by introducing various approximate methods. For example, conjugate GD [37], BFGS [38], and L-BFGS [39] are typical second-order methods used in the deep learning models.

Finally, various hybrid methods with local search algorithms have been widely utilized as the optimization methods to train diverse machine learning models. They aim to enhance the search ability of the existing optimization methods by combining the existing local search methods compensatively. For example, Yulian Cao and Han Zhang et al. proposed a comprehensive search method that uses particle swarm optimization and local search method simultaneously to solve multimodal problems [40]. Yuanyuan Tan and MengChu Zhou et al. proposed a hybrid scatter search method utilizing the mixed integer programming and scatter search methods to address the steelmaking-continuous casting problems [41]. Furthermore, Liliya A. Demidova and Artyom V. Gorchakov showed a novel hybrid optimization algorithm that combines merits of the collective behavior of fish schools and traditional first (or second)-order methods [42]. These study results indicates that the hybrid approach combining various strategies compensatively can significantly contribute to searching an approximate optimal solution in the complicated solution space.

### 3.3. Optimization Methods to Train CNNs

Since the residual connections have been introduced, the modern CNN models have more complicated architecture, i.e., deeper layers and more weights, than the traditional ones. Accordingly, a sophisticated and robust optimization algorithm is needed to train such large-scale CNN models effectively [43]. Among many first-order optimization methods for deep learning, Adam optimizer [18] is one of the most popular methods which have widely been utilized to train various neural networks. Algorithm 1 describes an overall architecture of Adam optimizer. Unlike the exiting methods such as GD and SGD, Adam optimizer utilizes the average search trajectories of the past gradients by introducing two momentums of the gradient, called the first and second momentums. In detail, the first momentum is the exponentially weighted average (EWA) of the past and current gradients. The second momentum is the EWA of the squared gradients, which is used to scale the first momentum. After the momentums are computed, these initial biases are corrected by the bias-correction method. Finally, the current weight $w_{t}$ is updated by the update rule of the GD shown in Equation (6). From Algorithm 1, we can find that the first momentum represents the most promising search direction determined by the past and current gradients. Moreover, the second momentum adjusts a strength of its movements toward the search direction with the learning rate α. Accordingly, Adam optimizer can perform more sophisticated solution search than those of the existing first-order optimization methods.
**Algorithm 1:** A pseudocode of Adam optimization algorithm **Algorithm: Adam** **Input:**
*f*, w, α, $\beta_{1}$, $\beta_{2}$ **Output:**
$w^{*}$ **Begin** Initialize $t=0,m_{1}=0,v_{1}=0$ **while** *not converged* **do:**
 t=t+1 $g_{t}=\nabla_{w}f\left(w_{t}\right)$ $m_{t+1}=\beta_{1}m_{t}+\left(1-\beta_{1}\right)g_{t}$ $v_{t+1}=\beta_{2}v_{t}+\left(1-\beta_{2}\right)g_{t}^{2}$ $m_{\hat{t}+1}=\frac{m_{t+1}}{1-\beta_{1}^{t}}$,   $v_{\hat{t}+1}=\frac{v_{t+1}}{1-\beta_{2}^{t}}$ $w_{t+1}=w_{t}-\alpha \frac{m_{\hat{t}+1}}{\sqrt{v_{\hat{t}+1}}}$ **end**
**while** $w^{*}=w_{t}$ **End** **Begin**

Accordingly, for the recent five years, diverse Adam-based optimization algorithms have been proposed. DiffGrad [23] proposed a friction method to decelerate its convergence to prevent that a CNN model is overfitted while it is being trained. Fromage [22] proposed a method to utilize the Euclidean distance to compute a difference between two gradients when computing the next search direction based on them. Meanwhile, TAdam [24] showed how to detect an outlier of gradients and estimate its coefficient adaptively when computing the first-momentum of gradients. For this, they derived the formulae to compute the first-momentum from a Student-t distribution using its maximum-likelihood estimation and utilized them to derive the first-momentum. Actually, they showed that TAdam had better optimization performance than the existing Adam-based optimizers.

Although such many methods to improve Adam optimizer have been studied, these still have difficulties in identifying the accurate and detailed information about their past and current gradients. In addition, they suffer from controlling a strength of their search elastically, which often makes finding an approximate optimal weight extremely hard. Despite of such difficulties, we aim to further improve these optimization performance by applying more sophisticated search control methods and adaptive coefficient computation methods. Accordingly, in the following section, we explain how our HyAdamC can effectively alleviate various problems from which the existing Adam-based optimization methods have suffered in training the CNNs in detail.

## 4. Proposed Method

### 4.1. Introduction

**Figure 3:**
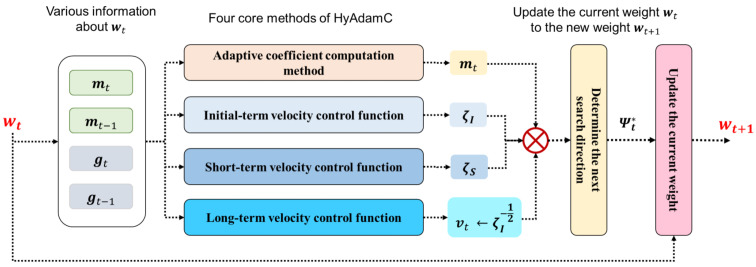
The overall architecture of HyAdamC and its hybrid mechanism.

In this section, we propose a novel Adam-based first-order optimization algorithm for training the CNNs effectively, called HyAdamC. Figure 3 illustrates an overall architecture of HyAdamC.

As shown in Figure 3, HyAdamC is composed of four core methods, i.e., the adaptive coefficient computation methods and three velocity control functions. The adaptive coefficient computation method calculates a coefficient of the first momentum adaptively depending on the difference between the past first momentum and current gradient to minimize an influence of any outlier gradients. The three velocity control functions, i.e., the initial-term, short-term, and long-term velocity control functions, adjust their search (convergence) velocity, i.e., a strength of search at each step, by considering the convergence states of the observed gradients.

Figure 3 also presents a hybrid mechanism of HyAdamC. Our intuition is to combine various strategies where each of them specializes in addressing each problem occurring in searching the optimal weights. In HyAdamC, the three velocity control function and the adaptive coefficient computation method are designed to alleviate each of various convergence problems. Then, they are coupled into one optimization method. In detail, first, the adaptive coefficient computation method is applied to compute the first-momentum at each step. Second, the first-momentum is divided by the long-term velocity control function to scale a strength of a search direction of the first-momentum. That is, the long-term velocity control function plays a role of the second-momentum in HyAdamC. Third, the initial-term and short-term velocity control functions are used to scale the first-momentum, which is implemented by multiplying them by the first-momentum. Then, the first-momentum scaled by the three velocity control functions is used as a next search direction. Accordingly, we can address diverse convergence problems comprehensively.

In the following subsections, we show how the three velocity control functions and the coefficient computation methods are implemented step by step. Then, their hybrid method is described with the detailed implementations.

### 4.2. Adaptive Coefficient Computation Method for the Robust First Momentum

**Figure 4:**
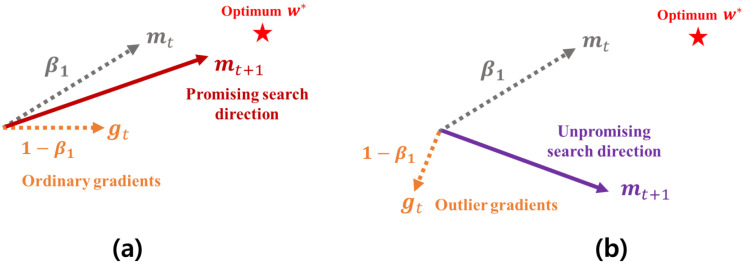
The example to explain how the outlier gradient negatively affects a direction the first momentum. (**a**) shows an ideal case that the first momentum is computed by the current momentum and the ordinary gradient. In this case, the first momentum becomes further close to the optimal weight. On the other hands, (**b**) shows a bad case that the first momentum is distorted by the unpromising (i.e., unexpected outlier) gradient. In this case, its next search direction moves away from the optimal weight by the unpromising gradient.

In the Adam-based optimizers, the next search direction is determined by the first momentum for the current gradient $g_{t}$, i.e., $m_{t}$. As demonstrated in Section 3, the first momentum maintains an average moving trajectory of its past gradients, which is computed by the EWA. At this time, if an unpromising gradient heading a direction far from the global optimum is found, a direction of the first momentum becomes further far from the approximate optimum, which makes its search ability seriously worse. Figure 4 shows how the first momentum is distorted by the unpromising gradients. As shown in Figure 4a, the next first momentum $m_{t+1}$ is computed by the EWA between $m_{t}$ and $g_{t}$ that are weighted by two constant coefficients $\beta_{1}$ and $\left(1-\beta_{1}\right)$, respectively. At this time, if $g_{t}$ heads an unpromising direction as shown in Figure 4b, a direction of $m_{t+1}$ is also skewed toward a direction of $g_{t}$. Thus, the next search direction gets further away from the approximate optimal weight $w^{*}$. From the example described in Figure 4, we can find that the existing method computing the first momentum is inherently vulnerable to these outlier gradients.

To overcome such shortcomings of the existing first momentum, it is required to check whether the current gradient is unpromising or not and reduce their influences as much as possible. For this, HyAdamC checks the difference between $m_{t}$ and $g_{t}$. If their difference becomes drastically large, a possibility that the direction of $g_{t}$ is unpromising is higher than their difference is small. In this case, we increase $\beta_{1}$ in proportion to a degree of their difference to minimize a force of $g_{t}$ in $m_{t+1}$. This mechanism is formulated as

(10) $$m_{t+1}=\beta_{1,t}m_{t}+\left(1-\beta_{1,t}\right)g_{t}$$

where $\beta_{1,t}$ is an adaptive coefficient defined by

(11) $$\beta_{1,t}\propto \left|m_{t}-g_{t}\right|.$$

In other words, $\beta_{1,t}$ determines a ratio of accumulation of $g_{t}$ in proportion to their difference. Actually, there exist many methods to implement Equation (11). Among them, we compute Equation (11) according to the methods shown in [24] as

(12) $$\beta_{1,t}=\frac{Q_{t-1}}{Q_{t-1}+q_{t}}.$$

In Equation (12), $q_{t}$ denotes a similarity between $g_{t}$ and $m_{t}$ that is measured by

(13) $$q_{t}=2d{\left(d+\sum_{j=1}^{d}\frac{{\left(g_{t},j-m_{t-1,j}\right)}^{2}}{v_{t-1,j}+\varepsilon }\right)}^{-1}$$

where $m_{t-1}$ and $v_{t-1}$ are the first and second momentums computed at the previous step, i.e., t−1. In addition, $Q_{t-1}$ in Equation (12) is a weighted sum of $q_{1},\cdots ,q_{t-1}$ that accumulates them as

(14) $$Q_{t-1}=\frac{2\beta_{1}-1}{\beta_{1}}Q_{t-2}+q_{t-1}.$$

Meanwhile, Equations (10)–(14) can be derived from the EWA of $g_{1},\cdots ,g_{t}$ by taking the partial differential of the normal distribution with respect to its μ. For this, we first apply the difference between $g_{t}$ and $m_{t}$ into the normal distribution. Second, we scale a random variable sampled from the modified normal distribution using the $\chi^{2}$-distribution. Third, we derive a new probability density function (PDF) from the scaled random variable. Finally, by taking a partial differential of the derived PDF with respect to μ, we can derive Equations (10)–(14). The detailed proofs for these are provided in the following Theorem 1.

**Theorem** **1.** *Let $N(\mu ,\sigma^{2})$ be a normal distribution and $\chi^{2}\left(d\right)$ be a $\chi^{2}$-distribution. In addition, let F(g;m,d) be a PDF derived by scaling a random variable sampled from N using $\chi^{2}$. Then, Equations (10)–(14) are derived from ∂F(g;m,d)/∂m.*

**Proof**  The detailed proofs are provided by “S.1. Proof of Theorem 1” in our supplementary file. □

**Figure 5:**
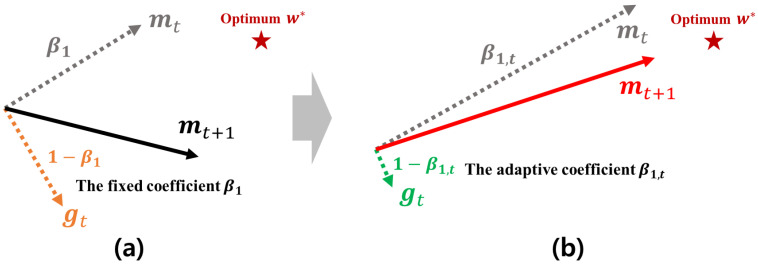
A brief mechanism of the first-momentum computation in HyAdamC. (**a**) describes a comparison of the existing first momentum computation method. (**b**) shows the new first momentum computation methods used in HyAdamC.

Figure 5 shows a brief mechanism of the first-momentum computation method used in HyAdamC. As described in Figure 5a, the existing first momentum uses a constant coefficient $\beta_{1}$. Accordingly, if $g_{t}$ heads an unpromising direction far from the optimum $w^{*}$, $m_{t+1}$ also proceeds to a direction of $g_{t}$. On the other hands, Figure 5b presents the first momentum computed by Equations (10)–(14) of HyAdamC. If the difference between $g_{t}$ and $m_{t}$ becomes large, Equation (12) also becomes increased. Accordingly, a coefficient of $g_{t}$, i.e., $1-\beta_{1,t}$ becomes low. Thus, $m_{t+1}$ heads a direction close to $m_{t}$ as an influence of $g_{t}$ is reduced as $1-\beta_{1,t}$. In other words, the outlier gradients far from the previous momentum have an influence as little as possible when the next first-momentum is computed. Therefore, we can effectively find a promising search direction which can access the optimal weight faster than the existing one.

### 4.3. Adaptive Velocity Control Functions

To find an approximate optimal weight $w^{*}$ effectively, it is necessary to control its search (or convergence) velocity elaborately depending on its past and current convergence states. For this, HyAdamC collects various information about the current optimization terrain from the past and current gradients. Then, these are utilized by the three adaptive control functions, i.e., the initial-term, short-term, and, long-term velocity control functions, to adjust the search velocity in various methods. In this section, we explain how HyAdamC controls its search velocity adaptively using the three velocity control functions.

#### 4.3.1. Initial-Term Velocity Control Function

In HyAdamC, the initial-term control function is designed to control a degree of its convergence at initial steps. In [44], the authors showed that controlling a strength of the convergence appropriately at initial steps could often help to improve the performance of the trained model. In order to implement such mechanism, they proposed a method that increases a strength of its convergence gradually as the step has been progressed, called *warm-up strategy*.

Accordingly, the initial-term velocity control function of HyAdam suppresses its search velocity at initial steps. Then, as the steps are progressed, this function fast increases its velocity. In detail, this function is formulated as

(15) $$\xi_{I}\left(\beta_{2},\rho_{t};\rho_{\infty }\right)={\left(\frac{\rho_{\infty }\left(1-{\beta_{2}}^{t}\right)\left({\rho_{t}}^{2}-6\rho_{t}+8\right)}{\rho_{t}\left({\rho_{\infty }}^{2}-6\rho_{\infty }+8\right)}\right)}^{\frac{\delta (\rho_{t})}{2}}$$

where $\beta_{2}$ is a coefficient used to compute the second momentum; $\rho_{\infty }=2/\left(1-\beta_{2}\right)-1$; $\rho_{t}=\rho_{\infty }-2t{\beta_{2}}^{t}{\left(1-{\beta_{2}}^{t}\right)}^{-1}$; $\delta (\rho_{t})$ is a Kronecker delta summation function defined by

(16) $$\delta \left(\rho_{t}\right)=1-\sum_{i=1}^{4}\delta_{\rho_{t},i}.$$

As shown in Equation (15), this function strongly suppresses a strength of its search to less than 0.1 at initial steps. Then, as the steps are progressed, its velocity becomes fast recovered because this function returns a value close to 1, which indicates that the search velocity is not suppressed by this function anymore. Therefore, we can control its convergence strength step by step by multiplying the first momentum $m_{t+1}$ computed by Equation (10) by Equation (15) when updating the weight $w_{t}$ to $w_{t+1}$. In Section 4.4, we will explain how this function is used with other functions in detail.

#### 4.3.2. Short-Term Velocity Control Function

**Figure 6:**
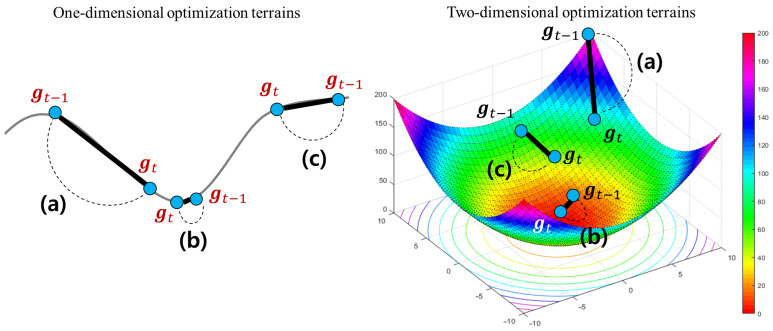
The examples to show a difference between the current gradient $g_{t}$ and its previous gradient $g_{t-1}$. The left figure shows that an one-dimensional loss function with several local minimums. The right figure describes a zoomed area around any local (or global) minimum of a two-dimensional loss function. In the two example plots, (**a**–**c**) illustrate three cases where the degree of variations between $g_{t}$ and $g_{t-1}$ is large, small, and medium, respectively.

The short-term velocity control function adjusts its search velocity depending on how much the current gradient $g_{t}$ has been changed when compared to the previous one $g_{t-1}$. For more convenient understanding, we assume that there are two example optimization terrains in one and two-dimensional solution spaces, respectively. Figure 6 illustrates three examples to explain what a difference between the previous and current gradients indicates on the optimization terrain.

As shown in Figure 6, the difference between $g_{t-1}$ and $g_{t}$ measures a degree of an instant variation from $g_{t-1}$ to $g_{t}$. This provides an useful information to understand the optimization terrain around $w_{t}$ in the solution space. For example, as shown in Figure 6b, if a degree of their instant variation is very small, we can guess that its current terrain is flatten. In this case, a possibility that there exists a local or global optimum around $w_{t}$ is relatively higher than other points. On the other hands, if a degree of the instant variation is large, as illustrated in Figure 6a, we can expect that the terrain around $w_{t}$ has been drastically changed. Finally, if two gradients have a gradual variation as described in Figure 6c, its convergence velocity has to be carefully adjusted to prevent too fast or slow searches. Thus, HyAdamC can check a current state of the terrain around $w_{t}$ by analyzing a degree of the instant variation from $g_{t-1}$ to $g_{t}$.

**Figure 7:**
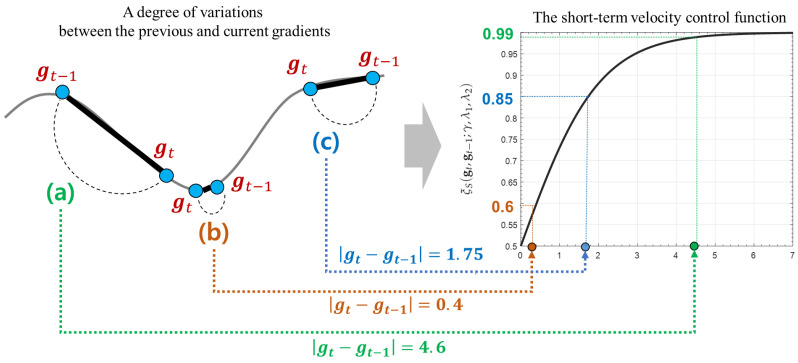
The principles of the short-term velocity control function. The right graph presents the plot of the short-term velocity control function described in Equation (17). (**a**) describes an example case that this function returns a value close to 1 as the difference between $g_{t}$ and $g_{t-1}$ becomes large. On the other hands, (**b**) shows other case that this function returns a value close to 0.5 as their difference becomes small. Finally, (**c**) illustrates how the short-term velocity control value is computed when a degree of their variation is 1.75. These examples show that this function can control the search velocity adaptively depending on a degree of variation of their previous and current gradients.

According to the principles explained above, the short-term velocity control function of HyAdamC is formulated as

(17) $$\xi_{S}\left(g_{t},g_{t-1};\lambda_{1},\lambda_{2}\right)={\left(1+e^{-\sigma_{t}^{\lambda_{1}}\left(\right|g_{t}-g_{t-1}\left|-\lambda_{2}\mu_{t}\right)}\right)}^{-1}$$

where $\lambda_{1}$ and $\lambda_{2}$ are model selection parameters; μ and σ indicate the mean and standard deviation of $|g_{t}-g_{t-1}|$, respectively. Figure 7 illustrates how an instant difference between $g_{t}$ and $g_{t-1}$ is mapped into the short-term velocity control function explained in Equation (17). As described in Figure 7a, if a degree of the instant variation from $g_{t}$ to $g_{t-1}$ is large, the search toward a direction of $g_{t}$ has to be encouraged because a possibility that there exist any steeply descending terrains around $w_{t}$ is high. Accordingly, this function returns a high value close to 1, which implies that its search velocity is not almost decreased. On the other hands, in Figure 7a, if these instant difference is very small, its search speed is strongly suppressed by a small value close to 0.5 because a possibility that there is an approximate optimal weight around $w_{t}$ is high. Accordingly, this function makes HyAdamC further carefully search around $w_{t}$. Similarly, Figure 7c illustrates that the search velocity when $|g_{t}-g_{t-1}|=1.75$ is decelerated as 0.85. Thus, the short-term velocity control function can effectively check a current state of its optimization terrain by analyzing a degree of the instant variation between the recent gradients and adjust a strength of its search velocity adaptively depending on the found terrain information.

Meanwhile, $\lambda_{1}\in \left\{0,1,2\right\}$ and $\lambda_{2}\in \left\{0,1\right\}$ in Equation (17) are used to determine how $|g_{t}-g_{t-1}|$ is scaled by μ and σ. For example, if $\lambda_{1}=0$ and $\lambda_{2}=0$, $|g_{t}-g_{t-1}|$ is not scaled by μ and σ. On the other hands, if $\lambda_{1}=\lambda_{2}=1$, it is scaled by them as $\sigma |g_{t}-g_{t-1}|-\mu$. Thus, we can implement six different models depending on these settings. Incidentally, the short-term velocity control function always returns a value between 0.5 and 1 as described in Figure 7. We can prove it as follows.

**Theorem** **2.** *The short-term velocity control function $\xi_{S}\left(g_{t},g_{t-1};\lambda_{1},\lambda_{2}\right)$ always returns a value between 0.5 and 1 according to the instant variation between any two recent gradients, i.e., $|g_{t}-g_{t-1}|$.*

**Proof**  This is proved by taking limit of Equation (17). Let $d_{i}=\left|g_{t}-g_{t-1}\right|$. Then, the limit of $\xi_{S}$ to zero is given by

(18) $$\lim_{d_{i}\to 0}\xi_{S}\left(g_{t},g_{t-1}\right)={\left(1+e^{\sigma_{t}^{\lambda_{1}}\lambda_{2}\mu_{t}}\right)}^{-1}.$$

In Equation (17), $\lambda_{1}\in \left\{0,1,2\right\}$ and $\lambda_{2}\in \left\{0,1\right\}$. In addition, $\mu_{t}\geq 0$ and $\sigma_{t}\geq 0$ because they are mean and standard deviation of $d_{i}$. Accordingly, we can find that $\sigma_{t}^{\lambda_{1}}\lambda_{2}\mu_{t}\geq 0$ and thus derive

(19) $$\lim_{d_{i}\to 0}\xi_{S}\left(g_{t},g_{t-1}\right)\geq 0.5.$$

Meanwhile, the limit of $\xi_{S}$ to *∞* is obviously 1 as

(20) $$\lim_{d_{i}\to \infty }\xi_{S}\left(g_{t},g_{t-1}\right)={\left(1+e^{-\infty }\right)}^{-1}=1.$$

Thus, $\xi_{S}\left(g_{t},g_{t-1}\right)$ returns a value between 0.5 and 1 according to $|g_{t}-g_{t-1}|$. □

#### 4.3.3. Long-Term Velocity Control Function

**Figure 8:**
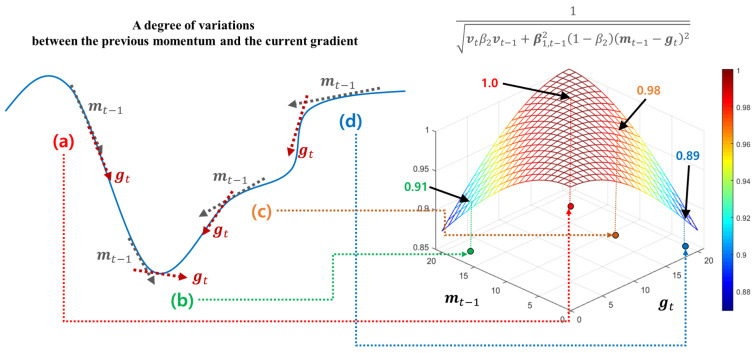
The principles of the long-term velocity control function. The left examples, i.e., (**a**–**d**) show how much the difference between $m_{t-1}$ and $g_{t}$. The right graph shows a plot of the long-term velocity control function in two-dimensional space. Each arrow from the left to the right figures describes how these differences are mapped to the long-term velocity control function.

Different to the short velocity control function utilizing a degree of variations between the previous and current gradients, the long velocity control function adjusts its search velocity by considering all the historical gradients, i.e., $g_{1}$, ⋯, $g_{t-1}$. For this, HyAdamC observes the previous first momentum $m_{t-1}$ to refer an average direction of the past gradients. Then, the difference between $m_{t-1}$ and $g_{t}$ is computed to control its search velocity adaptively. Figure 8 shows how these difference is used to adjust the search speed in HyAdamC.

Figure 8a,c show the cases where $g_{t}$ heads toward a similar direction to $m_{t-1}$. In this case, a possibility that a direction of $g_{t}$ is promising is further high because the current search direction is continuously similar to the previous one. Thus, as shown in the right graph of Figure 8, this function further enhances its search velocity by returning a value close to 1 to achieve faster convergence. On the other hands, Figure 8b,d describe another case that these difference is significantly large. Such cases imply that the search direction of $g_{t}$ has been drastically changed when compared to the previous average search direction, i.e., $m_{t-1}$. In this case, it is necessary to further carefully search the direction of $g_{t}$ because a possibility that the optimization terrain around $w_{t}$ has any steep slopes is relatively high. For this, this function reduces its convergence speed by returning relatively smaller values, to avoid hovering its search trajectory around $w_{t}$. Therefore, the long-term velocity control function is formulated as

(21) $$\xi_{L}\left(v_{t-1},m_{t-1},g_{t};\beta_{1},\beta_{2}\right)=\beta_{2}v_{t-1}+\beta_{1,t-1}^{2}\left(1-\beta_{2}\right){\left(m_{t-1}-g_{t}\right)}^{2}$$

where $\beta_{2}$ is a coefficient parameter to compute the EWA between $v_{t-1}$ and $g_{t}$. From Figure 8 and Equation (21), we can find that the long-term velocity control function is a momentum of $\beta_{1,t-1}^{2}{\left(m_{t-1}-g_{t}\right)}^{2}$. Accordingly, HyAdamC accumulates $\beta_{1,t-1}^{2}{\left(m_{t-1}-g_{t}\right)}^{2}$ using the EWA to utilize a degree of these long-term average variations in the next steps. Then, the long-term velocity control function is used as a new second momentum computation method instead of the existing second momentum shown in Algorithm 1 as

(22) $$v_{t+1}=\xi_{L}\left(v_{t},m_{t-1},g_{t};\beta_{1},\beta_{2}\right).$$

In the following sections, we explain how the three velocity control functions are used in the parameter update rule of HyAdamC. Furthermore, we show how our HyAdamC is implemented in detailed.

### 4.4. Parameter Update Methods and Implementations

To update the current weight $w_{t}$ to $w_{t+1}$, the next search direction at $w_{t}$ has to be determined from the first momentum. Let $\Psi_{t}$ be a bias-corrected first momentum, i.e.,

(23) $$\Psi_{t+1}=\frac{m_{t+1}}{1-\beta_{1}^{t}}.$$

Then, HyAdamC scales the bias-corrected first momentum $\Psi_{t+1}$ using the modified second momentum $v_{t+1}$, the learning rate α, and the three velocity control functions as

(24) $$\Psi_{t+1}^{*}=\frac{\xi_{I}\left(\beta_{2},\rho_{t}\right)\xi_{S}\left(g_{t},g_{t-1}\right)}{\sqrt{v_{t+1}}+\varepsilon }\Psi_{t+1}$$

where $v_{t+1}$ is a second momentum computed by the long-term velocity control function of Equation (22). Accordingly, $w_{t}$ is updated to $w_{t+1}$ by the first-order gradient descent method as

(25) $$w_{t+1}=w_{t}-\alpha \Psi_{t+1}^{*}$$

where α is a learning rate.

Algorithm 2 describes a complete implementation of our HyAdamC algorithm. At *t*th step, HyAdamC first gets the gradient of $w_{t}$, i.e., $\nabla g_{t}$. Next, the coefficient $\beta_{1,t}$ used in the first momentum $m_{t}$ is adaptively computed in proportion to the difference between the previous momentum $m_{t-1}$ and current gradient $g_{t}$. Then, the current first momentum $m_{t}$ is updated to $m_{t+1}$ by the EWA with $\beta_{1,t}$. At the same time, the second momentum $v_{t+1}$ is computed by the long-term velocity control function $\xi_{L}$.

After $m_{t+1}$ and $v_{t+1}$ are computed, the element used to compute $\beta_{1,t}$, i.e., $q_{t+1}$ is accumulated incrementally into $Q_{t+1}$ depending on Equation (14). Next, the initial biases of $m_{t+1}$ are corrected using $1-\beta_{1}^{t}$, which is equivalent method to that of Adam optimizer. Then, HyAdamC derives the next search direction $\Psi_{t+1}^{*}$ by scaling $m_{t+1}$ using $v_{t+1}$, $\xi_{I}$, and $\xi_{S}$, simultaneously. Accordingly, $w_{t}$ is updated to the new weight $w_{t+1}$ by the gradient descent rule. Finally, after any convergence condition is satisfied, the optimization process is terminated and the final approximate optimal weight $w^{*}$ is returned.
**Algorithm 2:** An implementation of HyAdamC**Algorithm: HyAdamC** **Input:***L*, w, α, $\beta_{1}$, $\beta_{2}$, $\lambda_{1}$, $\lambda_{2}$, ε **Output:** $w^{*}$ **Begin** $t=1,m_{1}=0,v_{1}=0,Q_{1}=0,g_{0}=0,w_{1}=w$ $\rho_{\infty }=\frac{2}{1-\beta_{2}}-1$ **while** *not converged* **do:** $g_{t}=\nabla_{w}L\left(f\left(x;w_{t}\right),y\right)$ $D=Dimensions(g_{t})$ $q_{t+1}=2D{\left(D+\sum_{j=1}^{D}\frac{{\left(g_{t,j}-m_{t,j}\right)}^{2}}{v_{t,j}+\varepsilon }\right)}^{-1}$ $\beta_{1,t}=\frac{Q_{t}}{Q_{t}+q_{t+1}}$ $m_{t+1}=\beta_{1,t}m_{t}+\left(1-\beta_{1,t}\right)g_{t}$ $v_{t+1}=\xi_{L}\left(v_{t},m_{t},g_{t}\right)$ $Q_{t+1}=\frac{2\beta_{1}-1}{\beta_{1}}Q_{t}+q_{t+1}$ $\Psi_{t+1}=\frac{m_{t+1}}{1-\beta_{1}^{t}}$ $\rho_{t}=\rho_{\infty }-\frac{2t\beta_{2}^{t}}{1-\beta_{2}^{t}}$ $\Psi_{t+1}^{*}=\xi_{I}\left(\beta_{2},\rho_{t}\right)\xi_{S}\left(g_{t},g_{t-1}\right)\frac{\Psi_{t+1}}{\sqrt{v_{t+1}}+\varepsilon }$ $w_{t+1}=w_{t}-\alpha \Psi_{t+1}^{*}$ t=t+1 **end** **while** $w^{*}=w_{t}$ **End** **Begin**

### 4.5. Regret Bound Analysis

In recent studies, the regret bounds of the Adam-based optimizers, such as Adam, diffGrad, and TAdam, have been analyzed by expanding the derivations of [45,46]. Actually, they provide significantly intuitive and useful methods to derive the regret bounds of the Adam-based optimizers. In addition, [24] showed more in-depth regret bound analysis methods in a case that an adaptive coefficient method is applied. Hence, we derived the regret bound of HyAdamC by utilizing the proofs of [24,45,46].

According to the methods of [24,45], several definitions are required to prove the upper regret bound of HyAdamC as follows. Let $w_{1},\cdots ,w_{T}$ ($\forall t,w_{t}\in F$) be the sequences found by HyAdamC and ${\hat{v}}_{1},\cdots ,{\hat{v}}_{T}$ be the sequence of the bias-corrected second momentums used in HyAdamC. In addition, let $\alpha_{t}=\alpha /t$, $\beta_{1,t}={\bar{\beta }}_{w}$, $\beta_{min}=min\left\{\beta_{1,1},\cdots ,\beta_{1,T}\right\}$, $\gamma ={\bar{\beta }}_{w}/\beta_{2}^{1/2}$, and $D_{\infty }$ is a bound diameter of *F*. Furthermore, a bounded gradients for a function $f_{t}$ is considered as $\forall t\in \left\{1,\cdots ,T\right\},w_{t}\in F$, $\|g_{t,w}{\|}_{2}\leq G$ and $\|g_{t,w}{\|}_{\infty }\leq G_{\infty }$ [23]. Then, the regret bound of HyAdamC is given as the following Theorem 3.

**Theorem** **3.** *Let $R_{T}$ be an upper regret bound of HyAdamC. Then, $R_{T}$ is given by*

$$\begin{array}{cc} R_{T} & \leq \frac{D_{\infty }^{2}}{\alpha_{T}\left(1-{\bar{\beta }}_{w}\right)}\sum_{i=1}^{d}\sqrt{{\hat{v}}_{T,i}}+\frac{D_{\infty }^{2}}{{\left(1-{\bar{\beta }}_{w}\right)}^{2}}\sum_{t=1}^{T}\sum_{i=1}^{d}\frac{\beta_{1,t}\eta_{S,t,i}^{-1}\sqrt{{\hat{v}}_{t,i}}}{\alpha_{t}\eta_{I,t}}\\ \; & \;\;+\frac{\alpha \sqrt{1+\log T}}{{\left(1-{\bar{\beta }}_{w}\right)}^{2}\left|\beta_{min}\right|\left(1-\gamma \right)\sqrt{\left(1-\beta_{2}\right)}}\sum_{i=1}^{d}{∥\begin{array}{c} g_{1:T,i} \end{array}∥}_{2}. \end{array}$$

**Proof.**  The detailed proofs are provided by “S.2. Proof of Theorem 3” in our supplementary file. □

We also can derive the average regret convergence of HyAdamC by utilizing a fact $\sum_{i=1}^{d}{\left\|g_{1:T,i}\right\|}^{2}\ll dG_{\infty }\sqrt{T}$ [23] and the results of Theorem 3. Let $\|g_{t,w}{\|}_{2}\leq G$ and $\|g_{t,w}{\|}_{\infty }\leq G_{\infty }$. Moreover, we assume that the weights found by HyAdamC satisfy $\|w_{n}-w_{m}{\|}_{2}\leq D$ and $\|w_{n}-w_{m}{\|}_{\infty }\leq D_{\infty },\forall n,m\in \left\{1,\cdots ,T\right\}$. Then, HyAdamC satisfies

(26) $$\frac{R(T)}{T}=O\left(\frac{1}{\sqrt{T}}\right),\forall T>1.$$

In other words, the upper regret bound of HyAdamC is converged to 0 as the step *t* increases from 1 to *T*. Then, we can understand a behavior of HyAdamC further concretely by taking the limit of Equation (26) as

(27) $$\lim_{T\to \infty }\frac{R(T)}{T}=0.$$

Thus, we can find that the the weights found by HyAdamC, i.e., $w_{1},w_{2},\cdots ,w_{T}$ become closer and closer to the optimal weight $w^{*}$ as the training steps are progressed.

## 5. Experiments

To evaluate the optimization ability of HyAdamC in practical CNN models, we performed various experiments with the SOTA CNN models and optimization methods. In Section 5.1, we first describe the overall experimental configurations. In Section 5.2, we explain the experiments conducted to choose the model of HyAdamC shown in Equation (17) and present these results. In Section 5.3, we evaluate the optimization performance of HyAdamC by comparing the image classification results of VGG, ResNet, DenseNet trained by HyAdamC with the results of other optimization methods and discuss these results in detail.

### 5.1. Experimental Settings

In this section, we explain the baseline CNN models, the compared optimization methods, benchmark datasets, and the experimental environment as follows.

**Figure 9:**
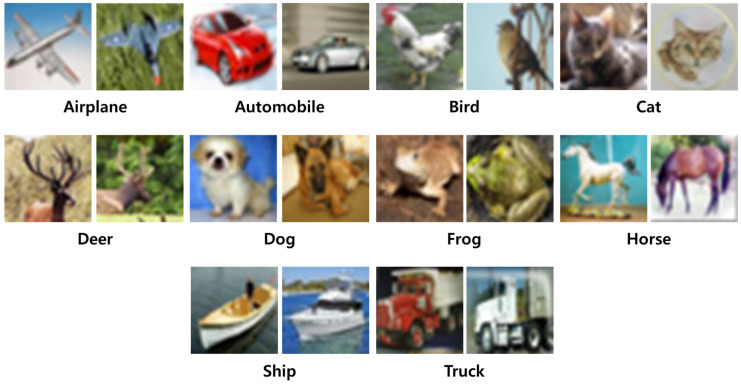
The example images in the CIFAR-10 dataset and their classes for image classification tasks.

- Compared optimization methods: As the optimization methods to compare the optimization performance of HyAdamC, we adopted 11 first-order optimization methods, i.e., SGD [16], Adam [18], RMSProp [36], AdaDelta [47], AdaGrad [20], AdamW [18], Rprop [48], Yogi [21], Fromage [22], diffGrad [23], and TAdam [24]. These have been extensively utilized to train various deep learning models involving the CNNs. Among them, in particular, Fromage, diffGrad, and TAdam are the latest optimization methods and have shown better optimization performance than the existing methods. In our experiments, all these parameters were set to the default values reported by their papers. The detailed parameter settings of our HyAdamC and the compared methods are listed in Table 1 (In all the methods except for RMSProp, their learning rate is denoted by α as shown in Algorithm 1).
- Baseline CNNs and benchmark datasets: In this experiments, VGG [12], ResNet [13], and DenseNet [14] were chosen as the baseline CNN models. In addition, we adopted the image classification task as a baseline task to evaluate the optimization performance of VGG, ResNet, and DenseNet models trained by HyAdamC and the compared methods. The image classification is one of the most fundamental and important tasks in the image processing applications and has been widely applied into many practical applications with the CNNs. Moreover, we adopted an universal benchmark image dataset, i.e., CIFAR-10 [49] image dataset which is one of the most popular benchmark datasets used to evaluate the image classification performance. In detail, the dataset involves 70,000 images with 60,000 training samples and 10,000 test ones. Each of them is a 32 × 32 color image and belongs to one of ten coarse classes. Figure 9 describes several example images and their classes briefly. In our experiments, the images involved in the dataset were utilized to evaluate how accurately the CNN models trained by HyAdamC and other algorithms could classify them.
- Metrics used in the experiments: In the experiments, we set the batch-size of the train/test samples to 64 and 128, respectively. The VGG, ResNet, and DenseNet were trained by HyAdamC and the compared methods. At this time, their weight values were randomly initialized by the default function provided in PyTorch. In detail, PyTorch initializes the weight value in each layer depending on the layer generation functions such as Linear() and Conv2d(). For example, the weights in Conv2d layers are initialized by Xavier method if any initialization method is not declared explicitly by a user. Different to ResNet and DenseNet, an implementation of VGG contains a function to initialize its weights. Thus, the weights of VGG were initialized by its initialization method. Furthermore, to maintain the same initial weight values for all compared methods, we fixed the random seeds of PyTorch, CUDA, and NumPy as a constant when training them. Then, we compared their performance and learning curves for the first 200 epochs. The training and test accuracies, i.e., $Acc_{train}$ and $Acc_{test}$ were measured by
  (28) $$Acc_{train}=\frac{\sum_{i=1}^{m_{train}}\delta_{y^{\left(i\right)},\hat{y^{\left(i\right)}}}}{m_{train}},$$
  (29) $$Acc_{test}=\frac{\sum_{i=1}^{m_{test}}\delta_{y^{\left(i\right)},\hat{y^{\left(i\right)}}}}{m_{test}}$$
  where $y^{\left(i\right)}$ is the GT class and $y^{\hat{\left(i\right)}}$ is the classified class for *i*th training sample, respectively; $\delta_{y^{\left(i\right)},y^{\hat{\left(i\right)}}}$ is a Kroneker delta; $m_{train}$ and $m_{test}$ are the total number of training and test image samples. In addition, the train and validation loss in our experiments were measured by the cross-entropy method. When the CNN model is defined as a K-classes classification problem, an cross-entropy loss for any ith input image is measured by
  (30) $$CE^{\left(i\right)}=-\sum_{j=1}^{K}t_{j}^{\left(i\right)}\log o_{j}^{\left(i\right)}$$
  where $t_{j}^{\left(i\right)}$ is the GT of the jth node and $o_{j}^{\left(i\right)}$ is an output value of the jth node for ith input image, respectively.
- Experimental environments: Our HyAdamC and other optimization methods were implemented and evaluated by Python 3.8.3 with PyTorch 1.7.1 and CUDA 11.0. In addition, we used matplotlib 3.4.1 library to represent our experimental results visually. Finally, all the experiments were performed on the Linux server with Ubuntu 7.5 OS, Intel Core i7-7800X 3.50GHz CPU, and NVIDIA GeForce RTX 2080Ti GPU.

### 5.2. Experiments to Choose the Convergence Control Model of Hyadamc

As shown in Equation (17), HyAdamC has two control options $\lambda_{1}$ and $\lambda_{2}$ which determine whether the mean and standard deviation of $|g_{t}-g_{t-1}|$ are used to scale it or not in the short-term velocity control function. Because $\lambda_{1}\in \left\{0,1,2\right\}$ and $\lambda_{2}\in \left\{0,1\right\}$, HyAdamC has six distinguished models according to these settings. Table 2 describes the six models of HyAdamC and the detailed formulae of their short-term velocity control functions.

Accordingly, we trained three CNN models, i.e., VGG, ResNet, and DenseNet, using the six HyAdamC models. Then, we compared their image classification results, i.e., test accuracies, to evaluate which model had the best optimization performance. Table 3, Table 4 and Table 5 describes the experiments results of VGG, ResNet, and DenseNet trained by HyAdamC-v1 to v6, respectively. First, Table 3 shows the test accuracies of the VGG-16 and 19 trained by HyAdamC-v1 to v6. In the experiments, HyAdamC-v1 and v5 achieved the first and second highest test accuracies in the 64 and 128-batched tests. In the tests for the ResNet-18 and 101 shown in Table 4, HyAdamC-v6 showed the best results for three of the four tests. In detail, the ResNet-18 trained by HyAdamC-v6 achieved 0.936 test accuracy in the 64-batched experiment and the ResNet-101 recorded 0.942 accuracy in both 64 and 128-batched tests. Meanwhile, as shown in Table 5, the DenseNet-121 and 169 trained by HyAdamC-v4 and V5 showed the best image classification performance. In particular, we found that HyAdamC-v5 achieved the first and second highest test accuracies in all four tests. From the results shown in Table 3, Table 4 and Table 5, we found that HyAdamC-v1, v3, v5, and v6 had relatively good optimization performance in VGG, ResNet, and DenseNet. On the other hands, HyAdamC-v2 and v4 showed slightly lower accuracies than others in the most experiments.

**Figure 10:**
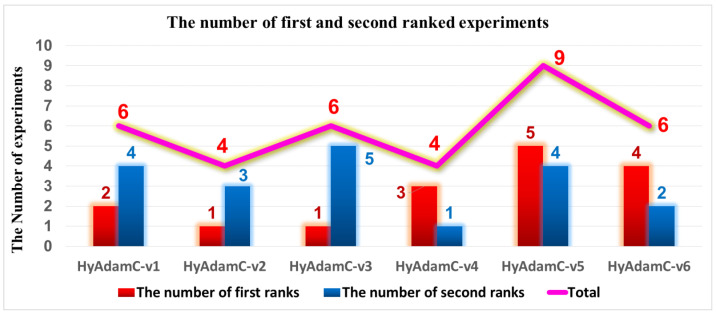
The number of the experiments in which HyAdamC-v1 to v6 achieved the first and second best test accuracies.

Meanwhile, Figure 10 describes the number of experiments in which each model achieved the first and second highest test accuracies. We found that HyAdamC-v5, v6, and v1 achieved the first, second, and third best results across all the experiments. In detail, HyAdamC-v5 recorded the first and second highest test accuracies in five and four experiments, respectively. HyAdamC-v6 showed the first and second best results in four and two experiments, respectively. Moreover, HyAdamC-v1 achieved the first and second best performance in two and four experiments, respectively. Accordingly, we chose HyAdamC-v1, v5, and v6 as the candidate models of HyAdamC by considering these results totally.

**Figure 11:**
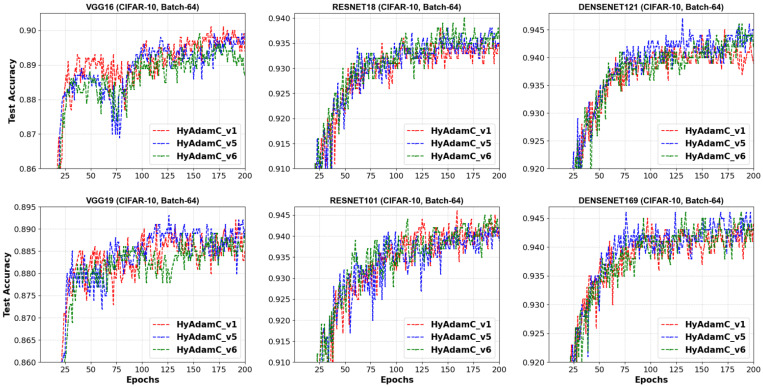
The test accuracy curves of VGG, ResNet, and DenseNet trained by HyAdamC-v1, v5, and v6 with CIFAR-10 images in the 64-batched experiments.

Additionally, we checked the convergence curves of the test accuracies recorded by HyAdamC-v1, v5, and v6. Figure 11 illustrates these test accuracy curves recorded in the 64-batched experiments. In VGG-16, the convergence curve of HyAdamC-v4 was relatively worse than that of HyAdamC-v1 around 75 steps. Nevertheless, as the epochs became progressed, HyAdamC-v5 gradually showed better accuracy than that of HyAdamC-v1. Moreover, HyAdamC-v6 showed relatively slower and lower convergence curves than others in both VGG-16 and 19. However, in ResNet-18 and 101, the its test curves were similar or slightly better than others. In the DenseNet-121 and 169, the test curves of HyAdamC-v5 presented better convergence than others.

**Figure 12:**
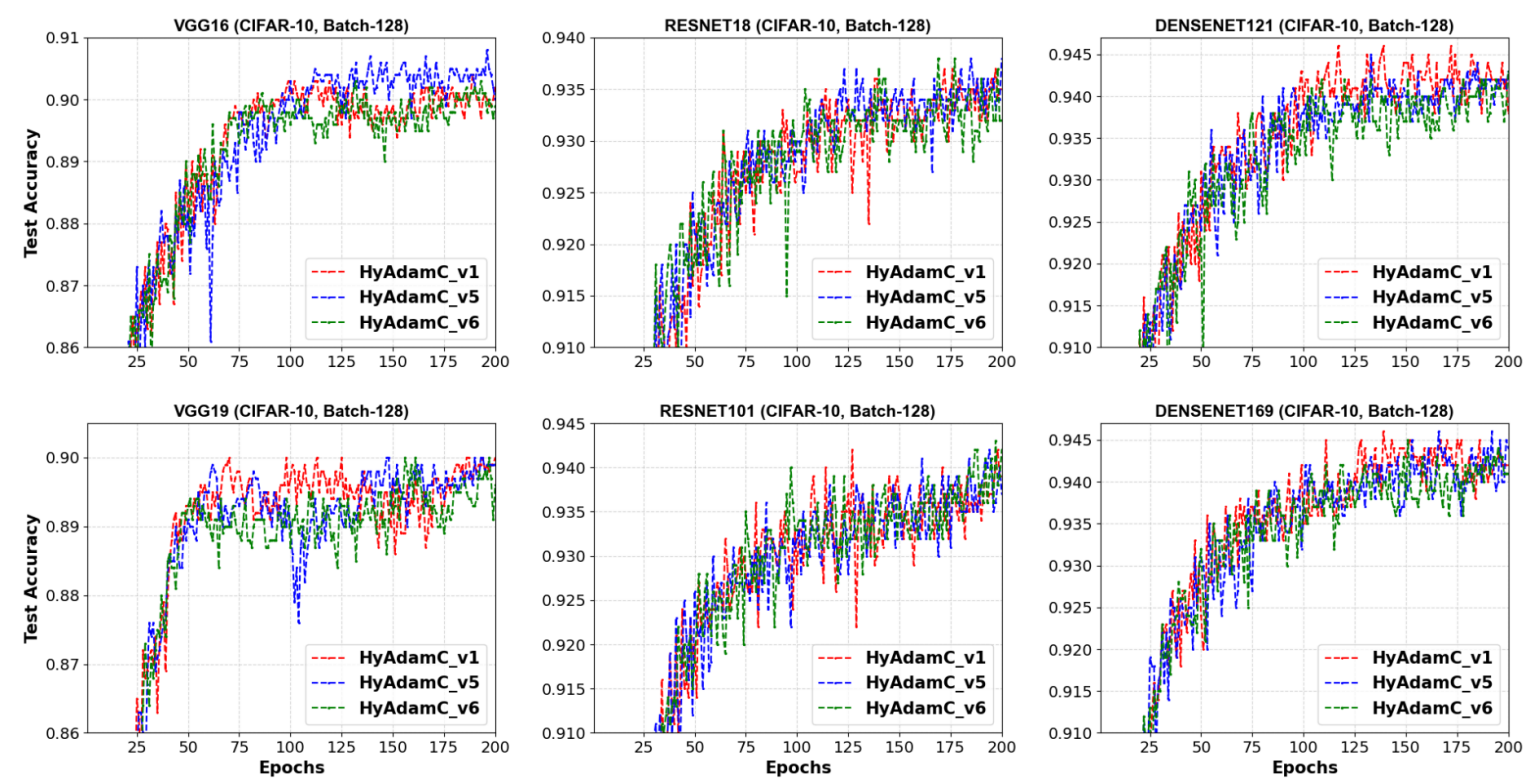
The test accuracy curves of VGG, ResNet, and DenseNet trained by HyAdamC-v1, v5, and v6 with CIFAR-10 images in the 128-batched experiments.

Figure 12 describes the test accuracies curves of HyAdamC-v1, v5, and v6 observed in the 128-batched tests. We found the HyAdamC-v1 had relatively stable convergence without notable oscillations in the tests except for the ResNet-101. Meanwhile, the test curves of HyAdamC-v5 and v6 showed slightly unstable convergence in the VGG-16, VGG-19, and ResNet-18. Interestingly, in the DenseNet-169, which is the most complicated model in our experiments, the three HyAdamC models showed significantly stable convergence with the high test accuracies. It indicates that our HyAdamC has stable and robust optimization ability for the complicated CNN models with a lot of layers.

In the experiments, HyAdamC-V1 showed most stable test convergence curves. On the other hands, HyAdamC-v5 finally achieved best test accuracies in the most experiments, even though it had slightly larger oscillations than those of HyAdamC-v1. Meanwhile, HyAdamC-v6 showed worse results than others in several models, particularly, VGG. Accordingly, we determined HyAdamC-v1 and HyAdamC-v5 as our final models. As explained in Table 2, HyAdamC-v1 is the baseline method that does not use the scale method. On the other hands, HyAdamC-v5 uses the variance to scale $|g_{t}-g_{t-1}|$ in Equation (17). Henceforth, we denote “HyAdamC-v1 and v5” as

- HyAdamC-v1 → *HyAdamC-Basic*,
- HyAdamC-v5 → *HyAdamC-Scale* (i.e., *scaled* HyAdamC)

with no confusions for simpler notations. Incidentally, we note that the configurations of $\lambda_{1}$ and $\lambda_{2}$ can be set variously depending on any characteristics of the CNN models or their applications.

### 5.3. Experimental Results in the Image Classification Tasks

#### 5.3.1. Image Classification Tasks in Vgg-16 and 19

Table 6 shows the test accuracies of VGG-16 and 19 trained by HyAdamC and other methods. The VGG-16 models trained by HyAdamC-Basic and HyAdamC-Scale achieved the best test accuracies among the compared methods. In detail, the VGG-16 and 19 trained by HyAdamC-Basic showed the best accuracies in 128-batched tests, i.e., 0.902 and 0.9 accuracies, respectively. On the other hands, in 64-batched tests, the VGG-16 and 19 trained by HyAdamC-Scale recorded 0.899 and 0.89 test accuracies, respectively, which were the highest results. Meanwhile, the VGG models trained by other methods showed relatively lower accuracies than those of HyAdamC-Basic and HyAdamC-Scale. In particular, RMSProp, AdamW, AdaDelta, and Yogi could not achieved promising training accuracies. It indicates that they could not train the VGG models normally. Such low training performance can occur by various causes such as inappropriate parameter settings and the vanishing gradient problem [19,35]. Such experimental results implies that the optimization methods are significantly sensitive to their parameter settings or any characteristics of the CNN models.

**Figure 13:**
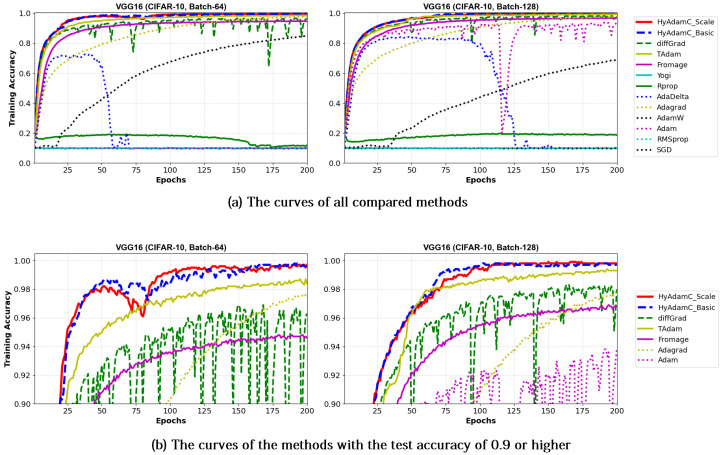
The training accuracy curves of the VGG-16 trained by HyAdamC and other optimization methods in the CIFAR-10 image classification tasks. In this figure, (**a**) shows the training accuracy curves of all compared methods. On the other hand, (**b**) illustrates the plots in which a range of the y-axis of the plots described in (**a**) is zoomed into between 0.9 and 1.

**Figure 14:**
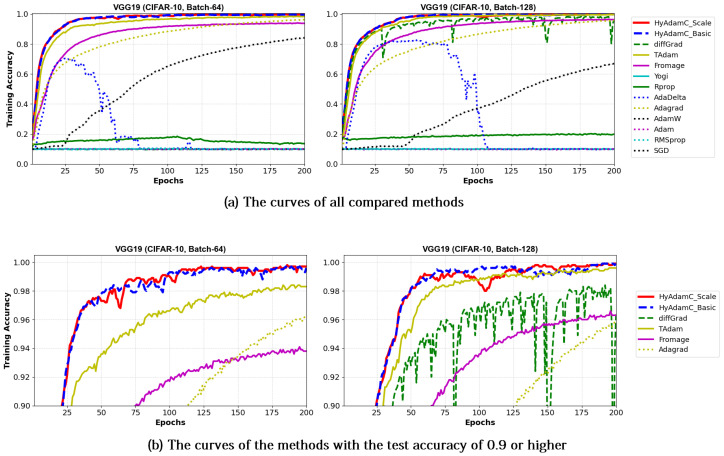
The training accuracy curves of the VGG-19 trained by HyAdamC and other optimization methods in the CIFAR-10 image classification tasks. In this figure, (**a**) shows the training accuracy curves of all compared methods. On the other hand, (**b**) illustrates the plots in which a range of the y-axis of the plots described in (**a**) is zoomed into between 0.9 and 1.

Figure 13 and Figure 14 show the training accuracy curves of the compared optimization methods in VGG-16 and 19, respectively. We found that VGG-16 and VGG-19 trained by HyAdamC-Basic and HyAdamC-Scale showed better training convergence than those of other methods. In particular, our curves showed considerably stable convergences when compared of those of other methods. Furthermore, other methods except for TAdam, Fromage, and AdaGrad, could not achieve reasonable training accuracies. As explained previously, these results indicates that the existing methods are significantly sensitive to the structural complexity of VGG. On the other hands, our HyAdamC-Basic and HyAdamC-Scale presented the most stable training convergence with the highest accuracies without notable oscillations even though the VGG was significantly vulnerable to the vanishing gradient problem.

**Figure 15:**
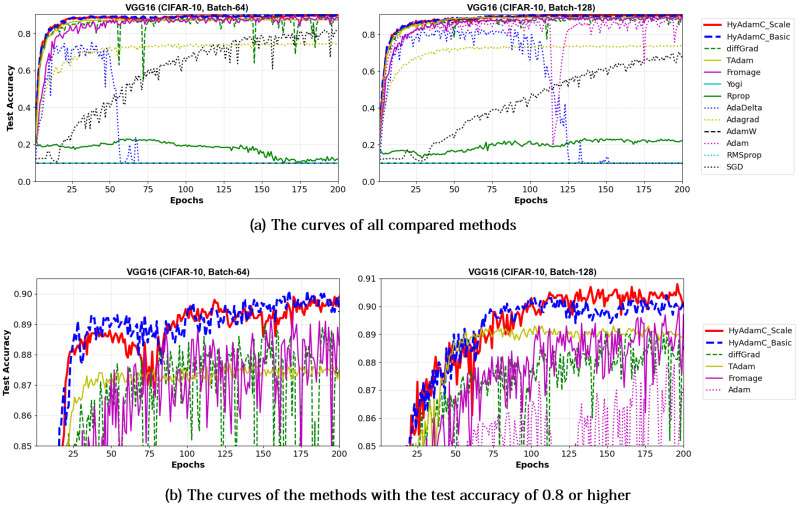
The test accuracy curves of the VGG-16 trained by HyAdamC and other optimization methods in the CIFAR-10 image classification tasks. In this figure, (**a**) shows the test accuracy curves of all compared methods. On the other hand, (**b**) illustrates the plots in which a range of the y-axis of the plots described in (**a**) is zoomed into between 0.85 and 0.9.

**Figure 16:**
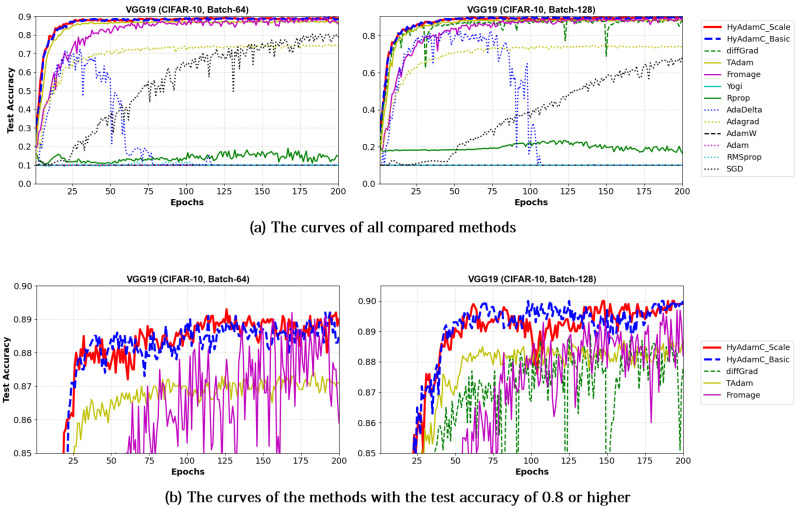
The test accuracy curves of the VGG-19 trained by HyAdamC and other optimization methods in the CIFAR-10 image classification tasks. In this figure, (**a**) shows the test accuracy curves of all compared methods. On the other hand, (**b**) illustrates the plots in which a range of the y-axis of the plots described in (**a**) is zoomed into between 0.85 and 0.9.

Meanwhile, Figure 15 and Figure 16 shows the convergence curves of their test accuracies in VGG-16 and 19, respectively. HyAdamC-Basic and HyAdamC-Scale also showed most robust and stable test convergence curves with the highest test accuracies. We notice that HyAdamC decelerates its search velocity slightly to control a strength of its convergence according to the warm-up strategy at initial steps. Furthermore, when a difference between the previous and current gradients is small, HyAdamC further reduces its search velocity to search around the current weights carefully in the solution space. Nevertheless, our HyAdamC achieved most stable not only the training but also test convergence curves with the best training/test accuracies. From the results, we found that HyAdamC had considerably robust and notable optimization performance for the CNN models that have suffered from the vanishing gradient problem.

#### 5.3.2. Image Classification Tasks in Resnet-18 and 101

Table 7 describes the test accuracies of the ResNet-18 and 101 trained by the HyAdamC and other methods. We found that the ResNet-18 and 101 trained by TAdam, diffGrad, and HyAdamC considerably improved their test accuracies when compared against other methods. Particularly, HyAdamC-Basic achieved the best accuracies in the three tests. In addition, the ResNet-101 trained by HyAdamC-Basic showed the best classification performance in both 64 and 128-batched experiments. Furthermore, the ResNet-18 trained by HyAdamC-Basic and HyAdamC-Scale also presented the best test accuracies in both 128 and 64-batched tests. Meanwhile, the ResNet-18 and 101 trained by TAdam and diffGrad also showed good test accuracies. In particular, diffGrad achieved better optimization performance than the result of the HyAdamC-Scale in the 128-batched test. Nevertheless, the test accuracies of both HyAdamC-Basic and HyAdamC-Scaled still were better than those of other methods.

**Figure 17:**
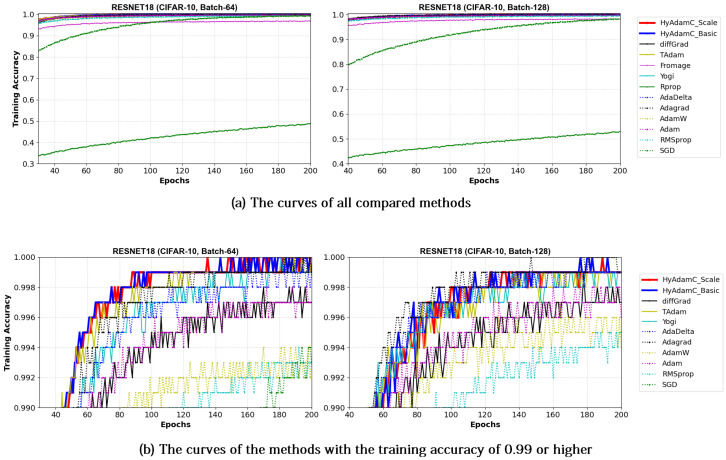
The training accuracy curves of the ResNet-18 trained by HyAdamC and other optimization methods in the CIFAR-10 image classification tasks. In this figure, (**a**) shows the training accuracy curves of all compared methods. On the other hand, (**b**) illustrates the plots in which a range of the y-axis of the plots described in (**a**) is zoomed into between 0.99 and 1.

**Figure 18:**
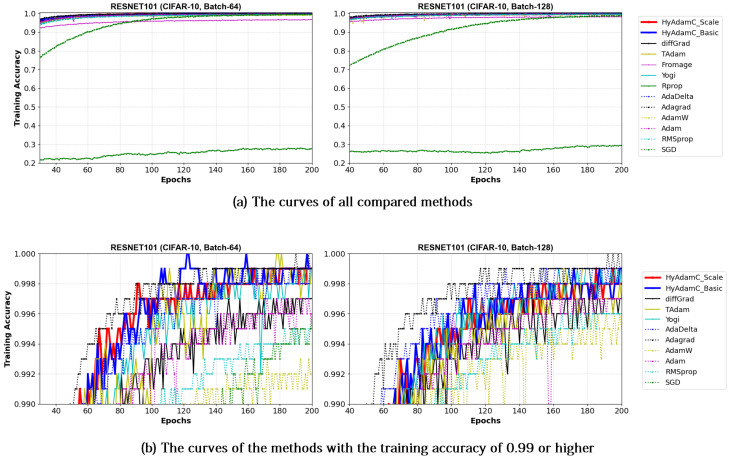
The training accuracy curves of the ResNet-101 trained by HyAdamC and other optimization methods in the CIFAR-10 image classification tasks. In this figure, (**a**) shows the training accuracy curves of all compared methods. On the other hand, (**b**) illustrates the plots in which a range of the y-axis of the plots described in (**a**) is zoomed into between 0.99 and 1.

Figure 17 and Figure 18 illustrate the training convergence curves of the ResNet-18 and ResNet-101, respectively. Different from the experiments for the VGG, most of the compared methods achieved high training accuracies more than 0.99. One of the reasons for such high training performance is that ResNet uses the residual connections, which is not used in VGG, to prevent the vanishing gradient problems [35,50]. As shown in Figure 17 and Figure 18, the ResNet-18 and 101 trained by HyAdamC showed overall similar or better training accuracies than others. Meanwhile, AdaGrad presented the fastest training convergence among all the compared methods including our HyAdamC. Nevertheless, the test accuracies of AdaGrad were lower than those of not only HyAdamC but also other SOTA methods such as TAdam and diffGrad. Such experimental results indicate that adjusting its training strength in the initial steps can contribute to improving its image classification abilities.

**Figure 19:**
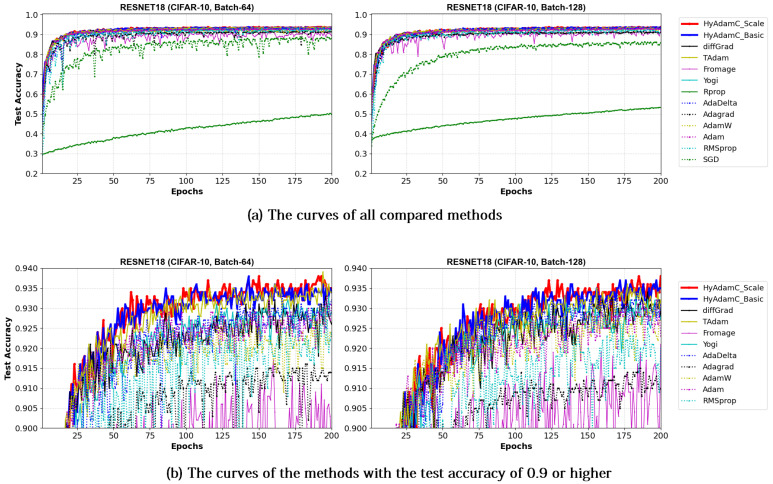
The test accuracy curves of the ResNet-18 trained by HyAdamC and other optimization methods in the CIFAR-10 image classification tasks. In this figure, (**a**) shows the test accuracy curves of all compared methods. On the other hand, (**b**) illustrates the plots in which a range of the y-axis of the plots described in (**a**) is zoomed into between 0.9 and 0.94.

**Figure 20:**
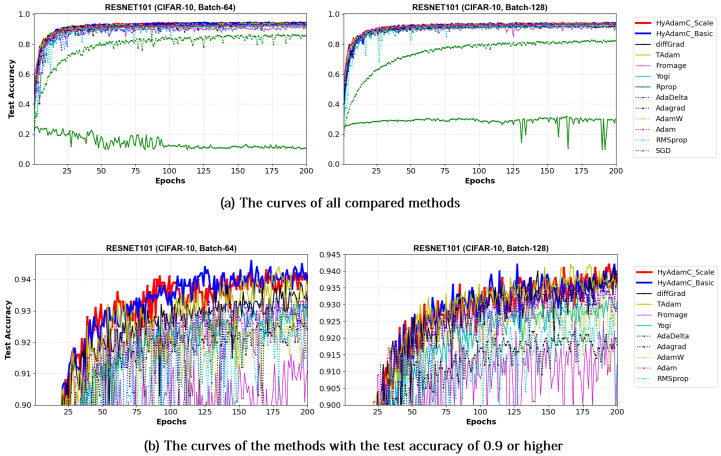
The test accuracy curves of the ResNet-101 trained by HyAdamC and other optimization methods in the CIFAR-10 image classification tasks. In this figure, (**a**) shows the test accuracy curves of all compared methods. On the other hand, (**b**) illustrates the plots in which a range of the y-axis of the plots described in (**a**) is zoomed into between 0.9 and 0.945.

Meanwhile, Figure 19 and Figure 20 show the convergence curves of test accuracies recorded by HyAdamC and other compared methods in the ResNet-18 and 101, respectively. We found HyAdamC-Basic and HyAdamC-Scale achieved overall better convergences than others. Meanwhile, from the 128-batched test shown in Figure 20, we found that the ResNet-101 trained by TAdam had similar or slightly better test accuracy convergence than those of HyAdamC. In particular, AdaGrad showed less test accuracy curves than those of HyAdamC-Basic and HyAdamC-Scale even though it had the fastest training convergence. As explained previously, because HyAdamC uses the warm-up strategy to control a degree of convergence at initial steps depending on the warm-up strategy. Accordingly, its initial training convergence can become slightly slower than others. Nevertheless, as the training has been progressed, its test accuracy becomes higher and higher with a faster ratio than others. Accordingly, HyAdamC can achieve better image classification performance than other methods.

#### 5.3.3. Image Classification Tasks in Densenet-121 and 169

As shown in Figure 2, the DenseNet constructs more residual connections between the inner blocks than that of ResNet. Accordingly, the DenseNet has more complicated architectures than those of the VGG and ResNet, which makes searching its optimal weights further hard.

Table 8 presents the test accuracies of the DenseNet-121 and 169 trained by HyAdamC and other methods. We found that the DenseNet models trained by HyAdamC outperformed other models in terms of the test accuracies. In detail, the DenseNet-121 trained by HyAdamC-Basic and HyAdamC-scale achieved the best test accuracies in all four tests. Particularly, HyAdamC-Scale showed the highest accuracies in three tests, i.e., 0.945 and 0.943 in the DenseNet-121 and 0.944 in the DenseNet-169, respectively. Meanwhile, the DenseNet-169 trained by HyAdamC-Basic showed the best test result in the 64-batched test, i.e., 0.944 test accuracy. In addition, it also achieved second highest test accuracies among all compared methods. Thus, HyAdamC-Basic and HyAdamC-Scale achieved the best test accuracies among all the compared methods, which is notable results when compared to other methods.

**Figure 21:**
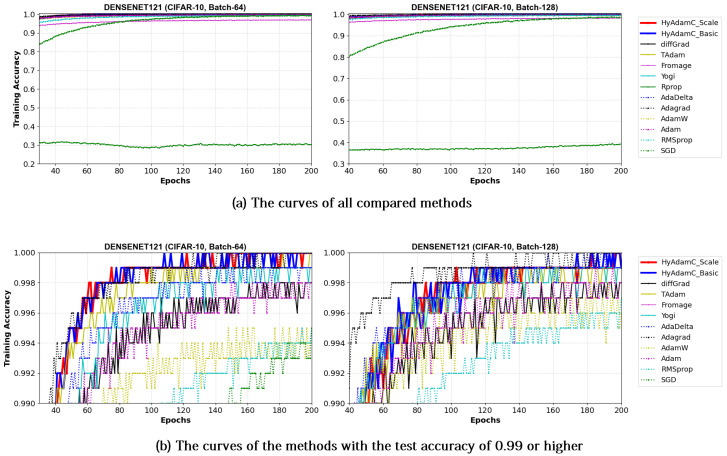
The training accuracy curves of the DenseNet-121 trained by HyAdamC and other optimization methods in the CIFAR-10 image classification tasks. In this figure, (**a**) shows the training accuracy curves of all compared methods. On the other hand, (**b**) illustrates the plots in which a range of the y-axis of the plots described in (**a**) is zoomed into between 0.99 and 1.

**Figure 22:**
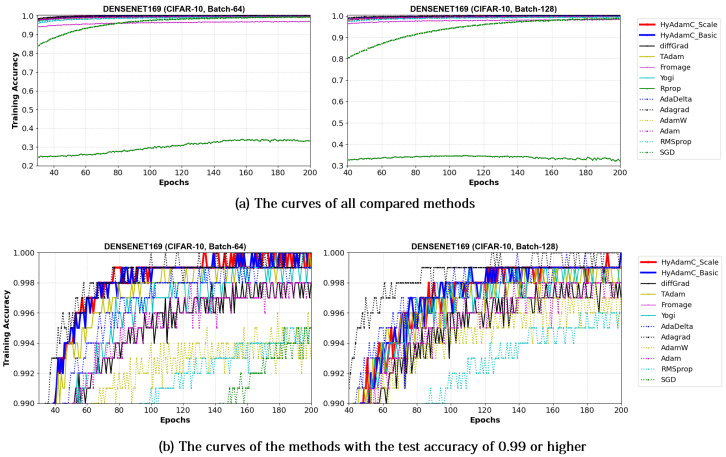
The training accuracy curves of the DenseNet-169 trained by HyAdamC and other optimization methods in the CIFAR-10 image classification tasks. In this figure, (**a**) shows the training accuracy curves of all compared methods. On the other hand, (**b**) illustrates the plots in which a range of the y-axis of the plots described in (**a**) is zoomed into between 0.99 and 1.

Figure 21 and Figure 22 illustrate the training accuracy curves in DenseNet-121 and 169 trained by HyAdamC and other methods, respectively. Similar to the results of ResNet, HyAdamC showed slightly slower convergence than those of AdaGrad. However, we also found that the HyAdamC-Basic and HyAdamC-Scale were further fast converged when compared to the curves of other methods. In particular, our HyAdamC still showed further stable and robust training accuracy curves when compared to ones in ResNet-18 and 101 even though the DenseNet-121 and 169 have more complicated architectures than the ResNet. On the other hands, other methods except for AdaGrad showed slower and lower convergence than those of HyAdamC-Basic and HyAdamC-Scale.

**Figure 23:**
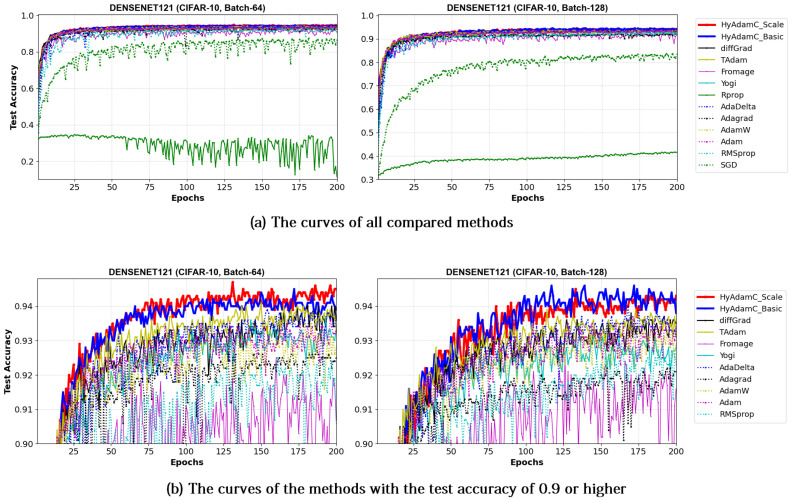
The test accuracy curves of the DenseNet-121 trained by HyAdamC and other optimization methods in the CIFAR-10 image classification tasks. In this figure, (**a**) shows the test accuracy curves of all compared methods. On the other hand, (**b**) illustrates the plots in which a range of the y-axis of the plots described in (**a**) is zoomed into between 0.9 and 0.95.

**Figure 24:**
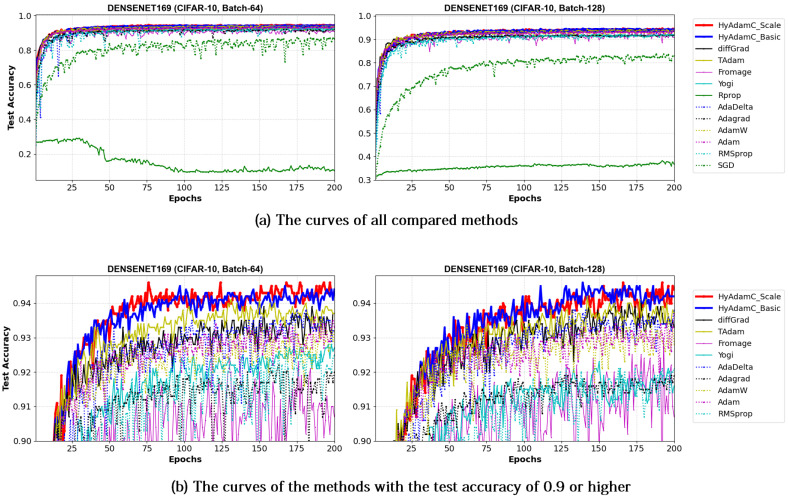
The test accuracy curves of the DenseNet-169 trained by HyAdamC and other optimization methods in the CIFAR-10 image classification tasks. In this figure, (**a**) shows the test accuracy curves of all compared methods. On the other hand, (**b**) illustrates the plots in which a range of the y-axis of the plots described in (**a**) is zoomed into between 0.9 and 0.95.

Such robust and stable training performance of HyAdamC-Basic and HyAdamC-Scale is also found in their test accuracy curves. Figure 23 and Figure 24 present that the DenseNet-121 and 169 trained by HyAdamC-Basic and HyAdamC-Scale showed significantly better test convergence curves with the highest test accuracies than those of other methods. In particular, we found that the gap between the curves of HyAdamC and other methods further widened when compared to the convergence curves of the ResNet-18 and 101 shown in Figure 19 and Figure 20. Furthermore, our test curves were significantly stable with no large-width oscillations. For example, in Figure 24, the test curves of Fromage and RMSProp showed considerably large oscillations. On the other hands, the test curves of HyAdamC-Basic and HyAdamC-Scale made the most stable and best convergences. In other words, as the CNN model was further complicated from ResNet to DenseNet, the optimization performance of other methods were slow down, however, HyAdamC-Basic and HyAdamC-Scale still maintained best test accuracies and stable convergence curves, simultaneously.

### 5.4. Additional Experiments to Evaluate the Performance of Hyadamc

#### 5.4.1. Image Classification Task in the Latest Lightweight Cnn Model: Mobilenet-V2

Different to VGG, ResNet, and DenseNet, MobileNet-v2 [15] is a lightweight CNN model designed to effectively perform image processing with a small amount of computations in limited environments such as mobiles or smart devices. Thus, MobileNet-v2 has relatively lightweight architecture when compared to ResNet and DenseNet. Accordingly, we conducted additional experiment to confirm how our HyAdamC behaves in the lightweight CNN models such as MobileNet-v2.

**Figure 25:**
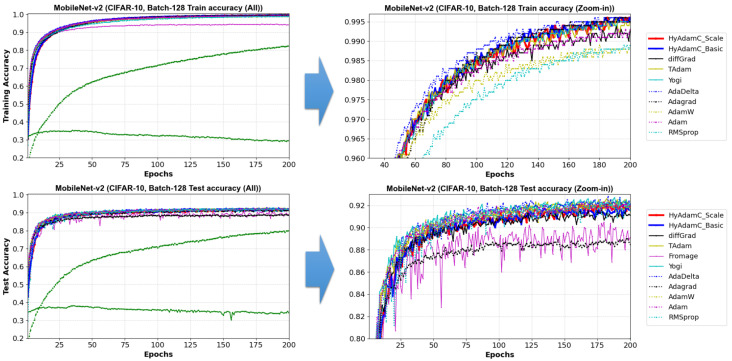
The training and test accuracy curves of the MobileNet-v2 trained by HyAdamC and other optimization methods in the CIFAR-10 image classification tasks. In this figure, the plots in the left side shows the training and test curves of all compared methods. On the other hand, ones in the right side illustrates the plots in which a range of the y-axis of the left plots is zoomed in.

Figure 25 describes training and test accuracy curves in MobileNet-V2. Different to the results in the VGG, ResNet, and DesNet, Figure 25 shows that the existing methods such as Adam and RMSProp achieved better test curves to those of HyAdamC. In particular, even though they showed comparable training curves when compared to the results of HyAdamC, their test curves were better than those of HyAdamC. Furthermore, the SOTA optimization methods such as Fromage and diffGrad also presented that worse test curves to those of HyAdamC and other traditional methods although they showed notable test accuracy in the ResNet and DenseNet. We think that such experimental results are caused by the lightweight architecture of MobileNet-V2. When compared to the traditional methods such as Adam and RMSProp, the SOTA methods involving our HyAdamC conducts further various operations to detect their search velocity in the complicated solution space. Accordingly, they perform more elastic search than the existing methods, which can cause relatively slower convergence than those of the existing methods.

To analyze the test accuracies of the compared methods in detail, we checked the their test accuracies at 200 epochs. Their results are listed in Table 9. As shown in Figure 25 and Table 9, HyAdamC-Basic and HyAdamC-Scale showed less test accuracies than the existing methods such as Adam, AdaDelta, and Yogi. TAdam, one of the SOTA methods, achieved equivalent to slightly better result than our HyAdamC. On the other hands, diff and Fromage showed less performance than HyAdamC even though they achieved high test accuracies in ResNet and DenseNet. Nevertheless, we also found that the difference in test accuracies between the methods that performed better than HyAdamC and HyAdamC was small. Such experimental results indicate that our HyAdamC can achieve comparable optimization performance in the lightweight CNN models.

#### 5.4.2. Validation Tests

**Figure 26:**
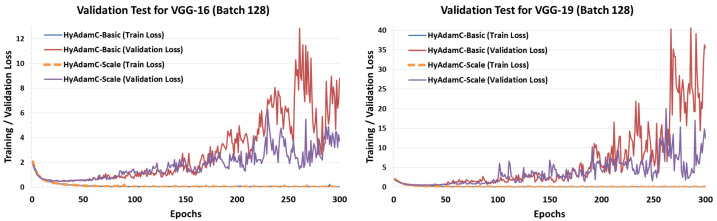
The training and validation loss curves of HyAdamC-Basic and HyAdamC-Scale in VGG. The left and right plots shows the loss curves of HyAdamC evaluated in VGG-16 and 19, respectively.

To confirm the number of suitable epochs of HyAdamC for each CNN models, we conducted validation tests. Figure 26 shows the training and validation loss curves of HyAdamC-Basic and HyAdamC-Scale in VGG-16 and 19, respectively. In the validation tests for VGG-16 and 19, we found that VGG-16 and 19 can be sufficiently learned by training less than about 50 times. In addition, it was found that the validation loss of HyAdamC was drastically increased when the epoch was performed more than 200 times. Furthermore, we found that HyAdamC-Scale showed more stable validation loss than HyAdamC-Basic. It indicates that the scale method using the variances in the short-term velocity function can contribute to making its optimization further stable.

**Figure 27:**
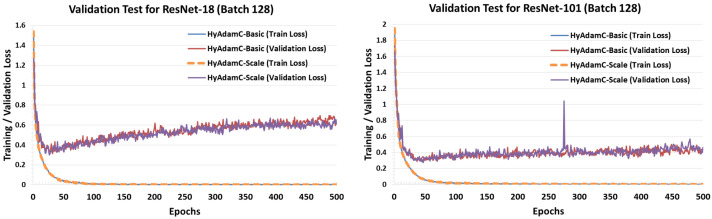
The training and validation loss curves of HyAdamC-Basic and HyAdamC-Scale in ResNet. The left and right plots shows the loss curves of HyAdamC evaluated in ResNet-18 and 101, respectively.

**Figure 28:**
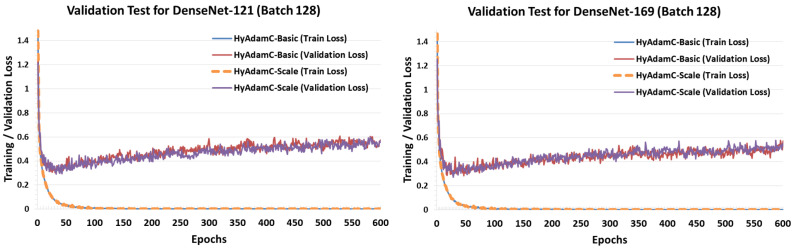
The training and validation loss curves of HyAdamC-Basic and HyAdamC-Scale in DenseNet. The left and right plots shows the loss curves of HyAdamC evaluated in DenseNet-121 and 169, respectively.

Figure 27 and Figure 28 describe the validation test results of HyAdamC in ResNet and DenseNet, respectively. Unlike the validation test results in VGG, both HyAdamC-Basic and HyAdamC-Scale showed stable validation curves in ResNet and DenseNet. In detail, we found that the validation losses in both ResNet and DenseNet were gradually increased after about 50 epochs were progressed. Also, when compared with the result of ResNet-18, we found that the validation loss curve was increased little by little as the epoch has been progressed. These results show that more epochs should be performed than those in ResNet-18 to sufficiently train ResNet-101.

**Figure 29:**
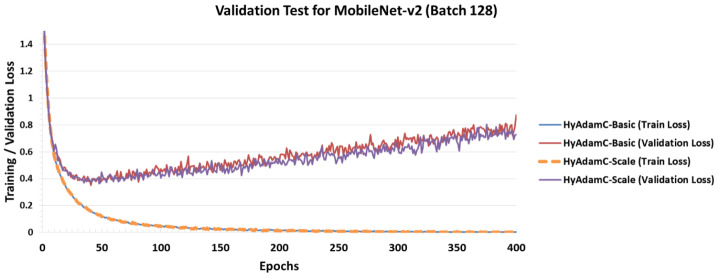
The training and validation loss curves of HyAdamC-Basic and HyAdamC-Scale in MobileNet-v2.

Figure 29 shows the training and validation loss curves of HyAdamC in MobileNet-v2. We found that the validation loss of MobileNet-v2 was increased after the epoch has been progressed more than about 50 times. In addition, both HyAdamC-Basic and HyAdamC-Scale were able to train MobileNet-v2 stably. The validation test results for the VGG, ResNet, and DenseNet imply that HyAdamC can train them effectively even with a relatively small number of epochs, depending on the complexity of their architecture in training the CIFAR-10 data set.

Meanwhile, when compared to the validation curves of Figure 26, Figure 27, Figure 28 and Figure 29, their test accuracy curves described in Section 5.3 and Section 5.4.1 show a phenomenon that their test accuracies were increased even though their validation losses were increased. Such phenomenon is caused by a difference of the methods that compute the test accuracy and validation loss. As shown in Equation (29), the test accuracy indicates a ratio of the number of correctly classified images among all the test images. On the other hands, the cross-entropy loss explained in Equation (30) is measured by computing an entropy of the real-valued output values of the nodes in the output layers. Therefore, when many sample images are used as the training, validation, and test sets and a number of training epochs are progressed, their test accuracy can be sometimes increased although their loss is increased according to their output values in the output layers.

#### 5.4.3. Image Segmentation in the U-Net

**Figure 30:**
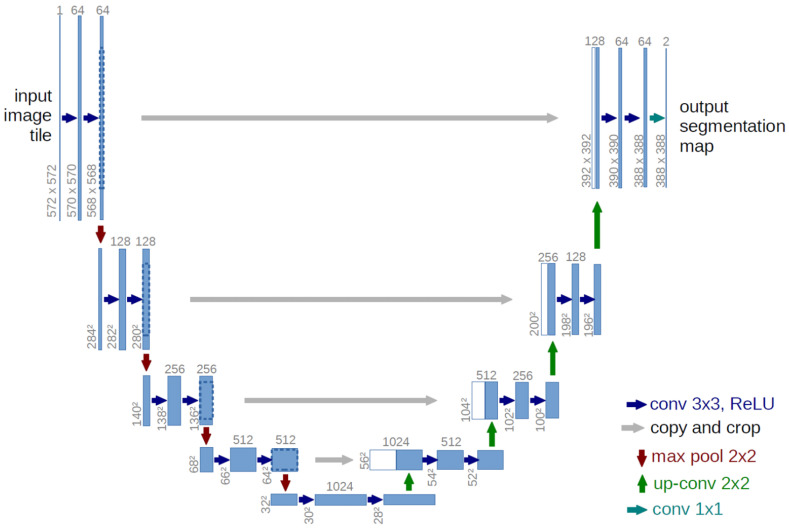
The basic architecture of the U-Net [51].

Finally, we designed another additional experiment to confirm whether HyAdamC can train not only the image classification models but also other applications performed by the CNNs. For this, we adopted the image segmentation task that identifies the semantic segments from any given images. In addition, we adopted the U-Net [51] as the baseline CNN model for image segmentation tasks. Figure 30 describes a basic architecture of the U-Net for image segmentation task.

**Figure 31:**
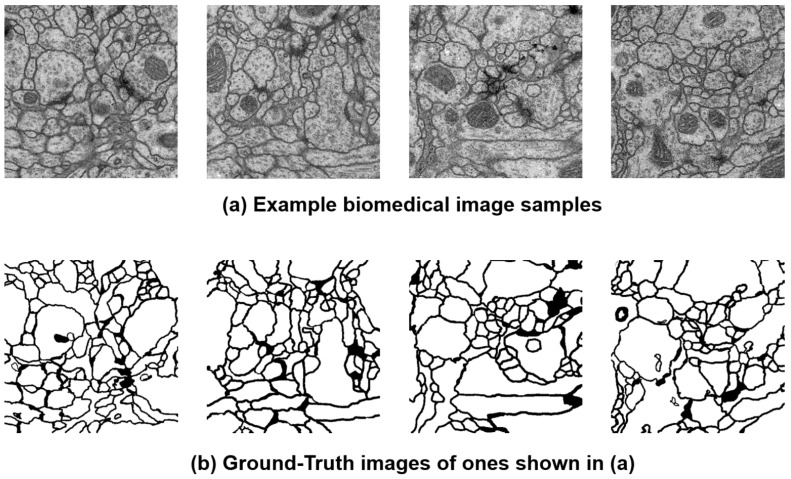
The example benchmark images involved in ssTEM dataset. (**a**) describes several sample training images and (**b**) illustrates their GT ones.

Our experiment goal is to confirm whether our HyAdamC can be applied to train a CNN model for the image segmentation. For this, the U-Net was trained by HyAdamC-Basic and HyAdamC-Scale using the biomedical images involved in the Transmission Electron Microscopy (ssTEM) dataset [52]. Figure 31 shows several example images used to train the U-Net. In our experiments, the number of epochs was set to 2000. In addition, the parameters of HyAdamC-Basic and HyAdamC-Scale were set to the default values shown in Table 1. Moreover, the cross entropy function was used as the loss function.

**Figure 32:**
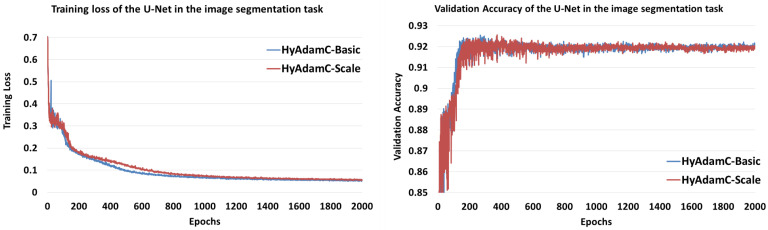
The training loss and validation accuracy curves of HyAdamC-Basic and HyAdamC-Scale in U-Net, respectively.

Figure 32 shows the training loss and validation accuracy curves of HyAdamC-Basic and HyAdamC-Scale, respectively. We found that HyAdamC-Basic had relatively faster training convergence than that of HyAdamC-Scale. Meanwhile, HyAdamC-Basic showed higher validation accuracies than those of HyAdamC-Scale at between 100 and 200 epochs. However, after about 800 epochs, the validation accuracies of HyAdamC-Basic were slightly better or equivalent than those of HyAdamC-Scale. Moreover, both HyAdamC-Basic and HyAdamC-Scale maintained stable validation accuracies with no notable increasing or decreasing after about 1000 epochs were progressed.

Table 10 lists their training losses and validation accuracies recorded at several epochs. After 2000 times epochs were progressed, their final validation accuracies were 0.92 and 0.919, respectively. In addition, they achieved the best validation accuracies, i.e., 0.9254 and 0.9256, at 267 and 373 epochs, respectively. Thus, we found that they had comparable performance in terms of the training losses and validation accuracies.

**Figure 33:**
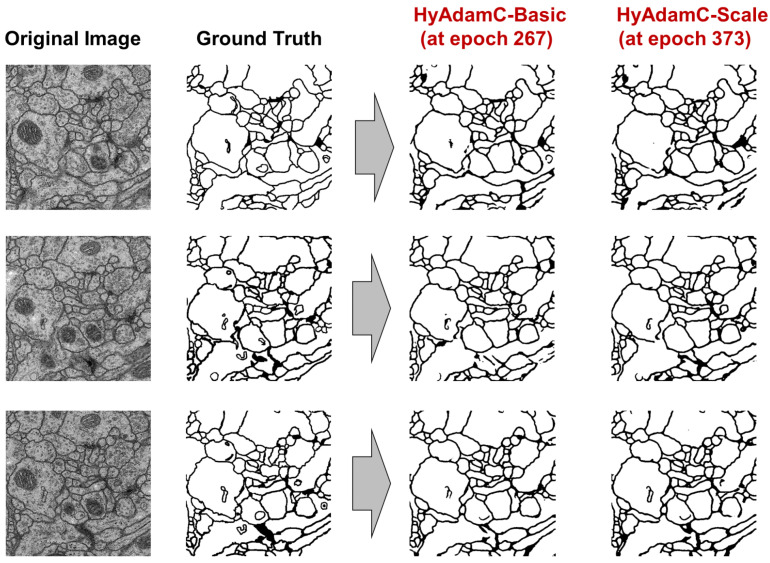
The images segmented by the U-Net trained by HyAdamC-Basic and HyAdamC-Scale. The left images show the original input images and their GT ones.

Finally, Figure 33 shows the validation results of U-Net trained by both HyAdamC-Basic and HyAdamC-Scale at epoch 267 and 373, respectively. As explained in Table 10, the most validation accuracies of HyAdamC-Basic and HyAdamC-Scale were 0.9254 and 0.9256, respectively. We found that the U-Net models trained by both HyAdamC-Basic and HyAdamC-Scale could effectively identify the segments from the input images when compared to their GT ones.

From these experimental results, we can find that our HyAdamC-Basic and HyAdamC-Scale have considerably robust and stable optimization performance regardless of the structural complexity of the CNN. Furthermore, we also found that our HyAdamC could be effectively applied not only the image classification but also image segmentation tasks.

## 6. Discussions

In this section, we briefly discuss our experimental results shown in Section 5 in terms of the three CNN models.

- VGG: As explained previously, the VGG is vulnerable to the vanishing gradient problem because it does not use the residual connections [13]. Accordingly, the existing methods such as SGD, AdaDelta, and Rprop showed considerably unstable convergence while training both the VGG-16 and 19 even though RMSprop and AdamW failed to train them. On the other hands, HyAdamC showed the most ideal convergence with the highest accuracies and the least oscillations when compared to other SOTA methods. It implies that our HyAdamC can perform considerably stable yet robust training even for the CNN models suffering from the vanishing gradient problems.
- ResNet: Different from the VGG, the ResNet alleviates the vanishing gradient problem by introducing the residual connections [13]. Accordingly, most of the compared methods, including HyAdamC, achieved high training accuracies 0.95 or higher in the experiments for ResNet-18 and 101. Nevertheless, HyAdamC still showed better test accuracies although it had slightly slower convergence than other SOTA methods. In particular, although AdaGrad presented faster convergence than HyAdamC, its test accuracies were significantly lower than those of HyAdamC. It indicates that the velocity control methods of HyAdamC are effective to search its optimal weight carefully on the complicated CNN models with the residual connections.
- DenseNet: As explained in Section 3, the DenseNet has more complicated architecture than the ResNet by constructing more residual connections [14]. Thus, the optimization terrain created in the DenseNet becomes further complicated than those in ResNet. In the experiments for the DenseNet-121 and 169, HyAdamC showed not only the best test accuracies but the most stable convergence. Especially, from Table 8, we found that the gap between HyAdamC and other methods became further increased when compared to the results in ResNet-18 and 101. Such results show the HyAdamC still maintains considerably robust and stable training ability with the highest accuracies in the complex architecture. Our velocity control functions and adaptive coefficient computation methods provide useful information about the complicated solution space of the DenseNet. Accordingly, HyAdamC could further elastically control its search strength and direction which helps to avoid falling into any local minimums or excessively oscillating around them. It is the most distinguished characteristic and merit of HyAdamC.

Thus, we found that HyAdamC had considerably robust and practical optimization abilities in training the CNNs. Such merits allow HyAdamC to be utilized to boost the performance of CNN models, e.g., the accuracy of image classification.

## 7. Conclusions

In this paper, we proposed a new Adam-based hybrid optimization algorithm, called HyAdamC. Our core intuition is to utilize various terrain information observed from the current and past gradients to effectively search its optimal weight. For this, HyAdamC exploits the three velocity control functions to elastically control its search velocity in terms of the initial, short, and long-term, respectively. Furthermore, HyAdamC effectively prevents that the first momentum is distorted by any outlier gradients by computing its coefficients adaptively according to a degree of variations of the past gradients.

In our experiments performed on the CIFAR-10 image classification tasks, the CNN models trained by HyAdamC showed better performance with stable training ability than those trained by the existing methods. It implies that the hybrid strategy of HyAdamC could contribute to enhancing its optimization performance, particularly, the image classification accuracy in the complicated CNN models such as ResNet and DenseNet. Furthermore, we also found that HyAdamC could be applied into not only image classification but also image segmentation tasks such as U-Net. Accordingly, in the near future, we will conduct more in-depth researches so that HyAdamC can be generally applied various models such as Recurrent Neural Networks (RNNs) [53] and Generative Adversarial Networks (GANs) [54].

## Figures and Tables

**Table 1:** The parameter settings of HyAdamC and the compared first-order optimization methods.

Algorithms	Parameter Settings
HyAdamC	$\alpha =10^{-3}$, $\beta_{1}=0.9$, $\beta_{2}=0.99$, $\varepsilon =10^{-8}$
SGD	$\alpha =10^{-3}$
RMSProp	Learning rate = $10^{-2}$, α=0.99, $\varepsilon =10^{-8}$
Adam	$\alpha =10^{-3}$, $\beta_{1}=0.9$, $\beta_{2}=0.99$
AdamW	$\alpha =10^{-3}$, $\beta_{1}=0.9$, $\beta_{2}=0.99$
Adagrad	$\alpha =10^{-2}$, $\beta_{1}=0.9$, $\varepsilon =10^{-10}$
AdaDelta	α=1.0, ρ=0.9, $\varepsilon =10^{-6}$
Rprop	$\alpha =10^{-2}$, $\eta_{-}=0.5$, $\eta_{+}=1.2$, step sizes $=[10^{-6},50]$
Yogi	$\alpha =10^{-2}$, $\beta_{1}=0.9$, $\beta_{2}=0.99$, $\varepsilon =10^{-3}$
Fromage	$\alpha =10^{-2}$
TAdam	$\alpha =10^{-3}$, $\beta_{1}=0.9$, $\beta_{2}=0.99$, v=d, $k_{v}=1.0$
diffGrad	$\alpha =10^{-3}$, $\beta_{1}=0.9$, $\beta_{2}=0.99$

**Table 2:** The six models of HyAdamC created by setting $\lambda_{1}$ and $\lambda_{2}$ in Equation (17).

Models	$\lambda_{1}$	$\lambda_{2}$	$\xi_{S}\left(g_{t},g_{t-1};\lambda_{1},\lambda_{2}\right)$
HyAdamC-v1	0	0	${\left(1+e^{-(|g_{t}-g_{t-1}|)}\right)}^{-1}$
HyAdamC-v2	0	1	${\left(1+e^{-(|g_{t}-g_{t-1}|-\mu_{t})}\right)}^{-1}$
HyAdamC-v3	1	0	${\left(1+e^{-\sigma_{t}\left(\right|g_{t}-g_{t-1}\left|\right)}\right)}^{-1}$
HyAdamC-v4	1	1	${\left(1+e^{-\sigma_{t}\left(\right|g_{t}-g_{t-1}\left|-\mu_{t}\right)}\right)}^{-1}$
HyAdamC-v5	2	0	${\left(1+e^{-\sigma_{t}^{2}\left(\right|g_{t}-g_{t-1}\left|\right)}\right)}^{-1}$
HyAdamC-v6	2	1	${\left(1+e^{-\sigma_{t}^{2}\left(\right|g_{t}-g_{t-1}\left|-\mu_{t}\right)}\right)}^{-1}$

**Table 3:** The test accuracies of the VGG-16 and 19 trained by the six HyAdamC models. The first and second best results are highlighted in red and orange, respectively.

	VGG-16		VGG-19	
Models	Batch 64	Batch 128	Batch 64	Batch 128
HyAdamC-v1	0.894	0.902	0.885	0.900
HyAdamC-v2	0.894	0.894	0.887	0.885
HyAdamC-v3	0.894	0.900	0.886	0.899
HyAdamC-v4	0.888	0.906	0.881	0.894
HyAdamC-v5	0.899	0.900	0.890	0.899
HyAdamC-v6	0.887	0.899	0.889	0.896

**Table 4:** The test accuracies of the ResNet-18 and 101 trained by the six HyAdamC models. The first and second best results are highlighted in red and orange, respectively.

	ResNet-18		ResNet-101	
Models	Batch 64	Batch 128	Batch 64	Batch 128
HyAdamC-v1	0.934	0.935	0.940	0.939
HyAdamC-v2	0.936	0.937	0.937	0.933
HyAdamC-v3	0.934	0.936	0.940	0.937
HyAdamC-v4	0.933	0.935	0.940	0.938
HyAdamC-v5	0.935	0.938	0.939	0.937
HyAdamC-v6	0.936	0.932	0.942	0.942

**Table 5:** The test accuracies of the DenseNet-121 and 169 trained by the six HyAdamC models. The first and second best results are highlighted in red and orange, respectively.

	DenseNet-121		DenseNet-169	
Models	Batch 64	Batch 128	Batch 64	Batch 128
HyAdamC-v1	0.939	0.938	0.944	0.942
HyAdamC-v2	0.942	0.938	0.943	0.942
HyAdamC-v3	0.943	0.940	0.942	0.945
HyAdamC-v4	0.941	0.943	0.944	0.942
HyAdamC-v5	0.945	0.943	0.943	0.944
HyAdamC-v6	0.942	0.943	0.943	0.941

**Table 6:** The test accuracies of the VGG-16 and 19 trained by the optimization methods for the CIFAR-10 image dataset classification task. “W (Win)”, “T (Tie)”, and “L (Loss)” refer to the number of the compared methods for which HyAdamC-Basic (or HyAdamC-Scale) achieved better, equivalent, and worse test accuracies, respectively. The first and second best results are highlighted in red and orange, respectively.

	VGG-16		VGG-19	
Methods	Batch 64	Batch 128	Batch 64	Batch 128
SGD	0.820	0.674	0.790	0.688
RMSProp	0.100	0.100	0.100	0.100
Adam	0.100	0.871	0.100	0.100
AdamW	0.100	0.100	0.100	0.100
Adagrad	0.746	0.738	0.740	0.742
AdaDelta	0.100	0.100	0.100	0.100
Rprop	0.123	0.223	0.149	0.166
Yogi	0.100	0.100	0.100	0.100
Fromage	0.883	0.897	0.859	0.882
TAdam	0.875	0.889	0.871	0.887
diffGrad	0.875	0.886	0.100	0.878
HyAdamC-Basic	0.894	0.902	0.885	0.900
HyAdamC-Scale	0.899	0.900	0.890	0.899
HyAdamC-Basic: W/T/L	11/0/0	11/0/0	11/0/0	11/0/0
HyAdamC-Scale: W/T/L	11/0/0	11/0/0	11/0/0	11/0/0

**Table 7:** The test accuracies of the ResNet-18 and 101 trained by the optimization methods for the CIFAR-10 image dataset classification task. “W (Win)”, “T (Tie)”, and “L (Loss)” refer to the number of the compared methods for which HyAdamC-Basic (or HyAdamC-Scale) achieved better, equivalent, and worse test accuracies, respectively. The first and second best results are highlighted in red and orange, respectively.

	ResNet-18		ResNet-101	
Methods	Batch 64	Batch 128	Batch 64	Batch 128
SGD	0.881	0.860	0.854	0.825
RMSProp	0.924	0.920	0.912	0.911
Adam	0.931	0.923	0.934	0.929
AdamW	0.923	0.927	0.922	0.928
Adagrad	0.914	0.910	0.924	0.918
AdaDelta	0.932	0.931	0.931	0.936
Rprop	0.498	0.533	0.102	0.302
Yogi	0.929	0.927	0.928	0.931
Fromage	0.894	0.921	0.911	0.912
TAdam	0.934	0.932	0.939	0.936
diffGrad	0.926	0.928	0.933	0.938
HyAdamC-Basic	0.934	0.935	0.940	0.939
HyAdamC-Scale	0.935	0.938	0.939	0.937
HyAdamC-Basic: W/T/L	10/1/0	11/0/0	11/0/0	11/0/0
HyAdamC-Scale: W/T/L	11/0/0	11/0/0	10/1/0	10/0/1

**Table 8:** The test accuracies of the DenseNet-121 and 169 trained by the optimization methods for the CIFAR-10 image dataset classification task. “W (Win)”, “T (Tie)”, and “L (Loss)” refer to the number of the compared methods for which HyAdamC-Basic (or HyAdamC-Scale) achieved better, equivalent, and worse test accuracies, respectively. The first and second best results are highlighted in red and orange, respectively.

	DenseNet-121		DenseNet-169	
Methods	Batch 64	Batch 128	Batch 64	Batch 128
SGD	0.865	0.835	0.866	0.830
RMSProp	0.906	0.923	0.925	0.921
Adam	0.933	0.934	0.933	0.937
AdamW	0.928	0.929	0.932	0.928
Adagrad	0.925	0.921	0.920	0.919
AdaDelta	0.937	0.931	0.932	0.938
Rprop	0.114	0.416	0.104	0.367
Yogi	0.933	0.928	0.927	0.916
Fromage	0.907	0.916	0.907	0.907
TAdam	0.933	0.931	0.937	0.937
diffGrad	0.936	0.935	0.937	0.933
HyAdamC-Basic	0.939	0.938	0.944	0.942
HyAdamC-Scale	0.945	0.943	0.943	0.944
HyAdamC-Basic: W/T/L	11/0/0	11/0/0	11/0/0	11/0/0
HyAdamC-Scale: W/T/L	11/0/0	11/0/0	11/0/0	11/0/0

**Table 9:** The test accuracies of the MobileNet-v2 trained by the optimization methods for the CIFAR-10 image dataset classification task. “*HyAdamC-Basic* −*Other Method*” (or “*HyAdamC-Scale*− *Other Method*”) indicates a difference between the test accuracies of HyAdamC-Basic (or HyAdamC-Scale) and the compared method. “W (Win)”, “T (Tie)”, and “L (Loss)” refer to the number of the compared methods for which HyAdamC-Basic (or HyAdamC-Scale) achieved better, equivalent, and worse test accuracies, respectively.

Methods	Test Accuracy	HyAdamC-Basic − Other Method	HyAdamC-Scale − Other Method
SGD	0.8	0.116	0.118
RMSProp	0.915	0.001	0.003
Adam	0.92	−0.004	−0.002
AdamW	0.914	0.002	0.004
Adagrad	0.885	0.031	0.033
AdaDelta	0.92	−0.004	−0.002
Rprop	0.341	0.575	0.577
Yogi	0.921	−0.005	−0.003
Fromage	0.891	0.025	0.027
TAdam	0.918	−0.002	0
diffGrad	0.911	0.005	0.007
HyAdamC-Basic	0.916	-	-
HyAdamC-Scale	0.918	-	-
Win/Tie/Lose (HyAdamC-Basic)			7/0/4
Win/Tie/Lose (HyAdamC-Scale)			7/1/3

**Table 10:** The detailed training losses and validation accuracies of HyAdamC-Basic and HyAdamC-Scale.

	HyAdamC-Basic		HyAdamC-Scale	
Epochs	Train Loss	Val.Acc.	Train Loss	Val.Acc.
50	0.3075	0.8694	0.3052	0.8790
100	0.2616	0.9027	0.2859	0.8901
200	0.1739	0.9233	0.1834	0.9223
500	0.0973	0.9199	0.1227	0.9222
1000	0.0684	0.9184	0.0753	0.9192
2000	0.0524	0.9200	0.0554	0.9190
	**Maximum Val. Acc.**	**Epochs**	**Maximum Val. Acc.**	**Epochs**
	0.9254	267	0.9256	373

## Data Availability

The CIFAR-10 dataset can be obtained from https://www.cs.toronto.edu/~kriz/cifar.html. The ssTEM dataset can be downloaded from ISBI Challenge: Segmentation of neuronal structures in EM stacks (http://brainiac2.mit.edu/isbi_challenge/home).

## Supplementary Materials

The following are available online at https://www.mdpi.com/article/10.3390/s21124054/s1. In the supplementary file, the detailed proofs of Theorem 1 and Theorem 3 are provided.

## Abbreviations

The following abbreviations are used in this paper:

CNN	Convolution neural network
CV	Computer vision
DenseNet	Densely connected network
DNN	Deep neural network
EWA	Exponentially weighted average
GAN	Generative adversarial network
GD	Gradient descent
GPU	Graphic processing unit
GT	Ground truth
IoT	Internet of Things
NLP	Natural language processing
PDF	Probability density function
ResNet	Residual network
RNN	Recurrent neural network
SGD	Stochastic gradient descent
SGDM	Stochastic gradient descent with momentum
SOTA	State-of-the-art
