# Supplementary material for: HyAdamC: A New Adam-Based Hybrid Optimization Algorithm for Convolution Neural Networks
Source: Sensors (Basel). 2021 Jun 12;21(12):4054. doi: 10.3390/s21124054 (PMC8231656; doi:10.3390/s21124054)
Supplement: Supplementary file 1 [file sensors-21-04054-s001.zip › sensors-1230374-supplementary.pdf]

HyAdamC: A New Adam-based Hybrid Optimization Algorithm for Convolution Neural Networks

- Supplementary files -

Kyung Soo Kim, Yong Suk Choi

June 11, 2021

S.1 Proof of Theorem 1

Let μ and σ^2 be the mean and variance of the normal distribution, respectively. Then, these sample mean and variance are defined as

$$\bar{x} = \frac{1}{n}(x_1 + ... + x_n),$$

$$s^2 = \frac{1}{n-1}\sum_{i=1}^{n}(x_i + ... + \bar{x})^2$$

where these are biased estimations of the ideal mean μ and variance σ^2. Now, we can scale the sample mean $\bar{x}$ to make it a random variable sampled from the standard normal distribution. Let W be a random variable sampled from the standard normal distribution and V be a random variable sampled from the χ^2-distribution. Then,

$$w = (\bar{x} - \mu)\frac{\sqrt{n}}{\sigma},$$

$$v = \frac{n-1}{\sigma^2}s^2$$

where the variance σ is eliminated by replacing σ to the sample standard deviation, i.e., s. We denote this modified random variable as

$$t = (\bar{x} - \mu)\frac{\sqrt{n}}{s} = \frac{w}{\sqrt{V/(n-1)}}$$

where $n-1$ is a degree of freedom which is defined as r. Therefore, we can derive the random variable in which the variance is eliminated as

$$t = \frac{w}{\sqrt{v/r}}$$

where $r = n - 1$.

Now, let $u = v$ for convenient proofs. Then, to derive the random variable that does not involve the variance, we first define a probability density function (PDF) $h(w, v)$ by multiplying the standard normal distribution by the χ^2-distribution as

$$h(w,v) = \frac{1}{\sqrt{2\pi}} e^{-w^2/2} \frac{1}{\Gamma(r/2)2^{r/2}} v^{r/2-1} e^{-v/2}$$

where $-\infty < w < \infty$ and $0 < v < \infty$. Then, a PDF for two random variables t and u is defined from $h(w, u)$ as

$$g(t,u) = h\left(\frac{t\sqrt{u}}{\sqrt{r}}, u\right)\sqrt{\frac{u}{r}}$$

where a PDF for t is derived from $g(t, u)$ by taking an integral with respect to u as

$$\begin{aligned} g_1(t) &= \int_{-\infty}^{\infty} g(t,u)du \\ &= \int_0^{\infty} \left(\frac{1}{\sqrt{2\pi r}\gamma(r/2)2^{r/2}} u^{(r+1)/2-1} e^{-\frac{u}{2}\left(1+\frac{t^2}{r}\right)}\right) du. \end{aligned}$$

For more concise expressions, let $z = u(1 + t^2/r)/2$. Then, this equation is simplified as

$$\begin{aligned} g_1(t) &= \int_0^{\infty} \left(\frac{1}{\sqrt{2\pi r}\Gamma(r/2)2^{r/2}} \left(\frac{2z}{1+t^2/r}\right)^{(r+1)/2-1} e^{-z}\left(\frac{2}{1+t^2/r}\right)\right) dz \\ &= \frac{\Gamma(\frac{r+1}{2})}{\sqrt{\pi r}\Gamma(r/2)} \frac{1}{(1+t^2/r)^{(r+1)/2}} \\ &= \frac{\Gamma(\frac{r+1}{2})}{\sqrt{\pi r}\Gamma(r/2)} \left(1+\frac{t^2}{r}\right)^{-\left(\frac{r+1}{2}\right)}. \end{aligned}$$

From this equation, we can find that this is a PDF of t-distribution with a degree of freedom r [2]. This distribution has been utilized to address various data that involves unknown variances or a few outliers [1, 5].

Meanwhile, we have to express this PDF as a multivariate form to utilize it in our HyAdamC. Accordingly, $g_1(t)$ is expressed as a d-dimensional multivariate form with n samples. Let $F(\mathbf{x})$ be a multivariate PDF of $g_1(t)$. Then, F is defined by

$$F(\mathbf{x}) = \prod_{i=1}^{n} \frac{\Gamma((r+d)/2)}{\Gamma(r/2)r^{d/2}\pi^{d/2}|\Sigma|^{1/2}} \left(1 + (\mathbf{x}_i - \boldsymbol{\mu})^T \boldsymbol{\Sigma}^{-1} (\mathbf{x}_i - \boldsymbol{\mu})\right)^{-(r+d)/2}.$$

From this equation, we can derive a log-likelihood function of F as follows.

$$
\begin{aligned}
\log F(\mathbf{x}) &= \sum_{i=1}^{n} \log \Gamma\left(\frac{r+d}{2}\right) - \sum_{i=1}^{n} \log \Gamma(r/2) r^{d/2} \pi^{d/2} |\Sigma|^{1/2} \\
&\quad - \frac{r+d}{2} \sum_{i=1}^{n} \log\left(1 + (\mathbf{x}_i - \boldsymbol{\mu})^T \boldsymbol{\Sigma}^{-1} (\mathbf{x}_i - \boldsymbol{\mu})\right) \\
&= n \log \Gamma\left(\frac{r+d}{2}\right) - n \log \Gamma\left(\frac{r}{2}\right) - \frac{dn}{2} \log r - \frac{dn}{2} \log \pi \\
&\quad - \frac{n}{2} \log |\Sigma| - \frac{r+d}{2} \sum_{i=1}^{n} \log(v + (\mathbf{x}_i - \boldsymbol{\mu})^T \boldsymbol{\Sigma}^{-1} (\mathbf{x}_i - \boldsymbol{\mu})).
\end{aligned}
$$

For further concise notations, let $K_i = (\mathbf{x}_i - \boldsymbol{\mu})^T \boldsymbol{\Sigma}^{-1} (\mathbf{x}_i - \boldsymbol{\mu})$. Then, the log-likelihood function $F(\mathbf{X})$ is simplified as

$$
\begin{aligned}
\log F(\mathbf{x}) &= n \log \Gamma\left(\frac{r+d}{2}\right) - n \log \Gamma\left(\frac{r}{2}\right) - \frac{dn}{2} \log r - \frac{dn}{2} \log \pi \\
&\quad - \frac{n}{2} \log |\Sigma| - \frac{r+d}{2} \sum_{i=1}^{n} \log(v + K_i)
\end{aligned}
$$

and its partial differential with respect to $\boldsymbol{\mu}$ is computed by

$$
\begin{aligned}
\frac{\partial}{\partial \boldsymbol{\mu}} F(\mathbf{x}) &= -\frac{r+d}{2} \sum_{i=1}^{n} (r+d) \frac{\partial \log(r + K_i)}{\partial \mu} \\
&= \sum_{i=1}^{n} \frac{(r+d)(x_i - \mu)}{r + K_i} \\
&= \sum_{i=1}^{n} \frac{(r+d)x_i - (r+d)\mu}{r + K_i}.
\end{aligned}
$$

By setting this equation to 0, we can derive a formula to compute a mean of F as

$$
\begin{aligned}
\mu \sum_{i=1}^{n} \frac{r+d}{r+K_i} &= \sum_{i=1}^{n} \frac{r+d}{r+K_i} x_i \\
\therefore \hat{\mu}_n &= \left(\sum_{i=1}^{n} \frac{r+d}{r+K_i}\right)^{-1} \sum_{i=1}^{n} \frac{r+d}{r+K_i} x_i.
\end{aligned}
$$

In the above equation, let $q_i = (r+d)/(r+K_i)$ and $Q_n = \sum_{i=1}^{n} q_i$. Then, this equation becomes further simplified as

$$
\hat{\mu}_n = Q_n^{-1} \sum_{i=1}^{n} x_i q_i
$$

where the nominator and denominator can be represented as the incremental formulae using q_i and Q_n as follows.

$$\begin{aligned}\hat{\mu}_n &= \frac{1}{Q_{n-1}+q_n}\left(\sum_{i=1}^{n-1} x_i q_i + x_n q_n\right)\\ &= \frac{Q_{n-1}}{Q_{n-1}+q_n}\hat{\mu}_{n-1} + \frac{q_n}{Q_{n-1}+q_n}x_n\\ &\because \hat{\mu}_{n-1} = Q_{n-1}^{-1}\sum_{i=1}^{n-1} x_i q_i.\end{aligned}$$

Thus, $\hat{\mu}_n$ is a typical exponential weighted average where the observed values are $x_1, ..., x_n$ and the coefficients are $Q_{n-1}/(Q_{n-1}+q_n)$ and $q_n/(Q_{n-1}+q_n)$. In particular, we can find that $\hat{\mu}_n$ is equivalent to the first momentum $\mathbf{m}_t$ and $Q_{n-1}/(Q_{n-1}+q_n)$ is mapped to $\beta_{1,n}$. Thus, the first momentum computation formula is expressed as

$$\begin{aligned}\mathbf{m}_{t+1} &= \boldsymbol{\beta}_{1,t}\mathbf{m}_t + (1-\boldsymbol{\beta}_{1,t})\mathbf{g}_t\\ &= \frac{Q_t}{Q_t+q_{t+1}}\mathbf{m}_t + \frac{q_t}{Q_t+q_{t+1}}\mathbf{g}_t.\end{aligned}$$

Meanwhile, we can improve $Q_{t+1} = Q_t + q_{t+1}$ by using a weighted decay [3]. For this, a new coefficient ς is introduced as

$$\varsigma = \frac{2\beta_1 - 1}{\beta_1}.$$

Then, Q_{t+1} is computed by

$$Q_{t+1} = \varsigma Q_t + q_{t+1} = \frac{2\beta_1 - 1}{\beta_1}Q_t + q_{t+1}.$$

In addition, the degree of freedom r can be set to d (i.e., $r = d$) because the first momentum of HyAdamC addresses n-dimensional gradients. Besides, $K_i = (\mathbf{x}_i - \boldsymbol{\mu})^T\boldsymbol{\Sigma}^{-1}(\mathbf{x}_i - \boldsymbol{\mu})$ can be also modified by approximating Σ to the second momentum $\mathbf{v}$ according to the method proposed by [3]. In other words, K_i is expressed as

$$K_i = (\mathbf{x}_i - \boldsymbol{\mu})^T\mathbf{v}^{-1}(\mathbf{x}_i - \boldsymbol{\mu}).$$

Accordingly, we can finally express the formulae to compute the adaptive first momentum in HyAdamC as

$$
\begin{aligned}
K_t &= \sum_{j=1}^{d} \frac{(\mathbf{g}_{t,j} - \mathbf{m}_{t,j})^2}{\mathbf{v}_{t,j} + \varepsilon} \\
q_{t+1} &= \frac{2d}{d + K_t} \\
\boldsymbol{\beta}_{1,t} &= \frac{Q_t}{Q_t + q_{t+1}} \\
\mathbf{m}_{t+1} &= \frac{Q_t}{Q_t + q_{t+1}} \mathbf{m}_t + \frac{q_t}{Q_t + q_{t+1}} \mathbf{g}_t \\
Q_{t+1} &= \varsigma Q_t + q_{t+1}
\end{aligned}
$$

where $\mathbf{m}_t$ and $\mathbf{v}_t$ are the first and second momentums computed at the previous step $t-1$. Thus, we have derived the formulae used in Section 4.2.

S.2 Proof of Theorem 3

In 2018, the authors of [4] showed how to derive the regret bound of the Adam-based optimization methods that can be utilized as a baseline to analyze the regret bound of HyAdamC. Furthermore, the authors of [3] proved the upper regret bound of TAdam by referring [4] and [6]. Thus, in this proof, we utilize the proofs of [3] and [4] to derive the regret bound of HyAdamC.

Let η_t be a multiplication of the initial and short-term velocity control functions, i.e.,

$$
\eta_t = \zeta_{I,t} \zeta_{S,t} = \left(\frac{\rho_\infty (1 - {\beta_2}^t)({\rho_t}^2 - 6\rho_t + 8)}{\rho_t ({\rho_\infty}^2 - 6\rho_\infty + 8)} \right)^{\frac{\delta(\rho_t)}{2}} \left(1 + e^{-\boldsymbol{\sigma}_t^{\lambda_1} (|\mathbf{g}_t - \mathbf{g}_{t-1}| - \lambda_2 \boldsymbol{\mu}_t)} \right)^{-1}.
$$

In this equation, we set $\lambda_1 = 0$ and $\lambda_2 = 0$ to derive the regret bound of the HyAdamC-Basic which is a baseline model of HyAdamC. Then, η_t is simplified as

$$
\eta_t = \left(\frac{\rho_\infty (1 - {\beta_2}^t)({\rho_t}^2 - 6\rho_t + 8)}{\rho_t ({\rho_\infty}^2 - 6\rho_\infty + 8)} \right)^{\frac{\delta(\rho_t)}{2}} \left(1 + e^{-|\mathbf{g}_t - \mathbf{g}_{t-1}|} \right)^{-1}
$$

which is utilized to derive the upper regret bound of our HyAdamC in the following proofs.

Meanwhile, the upper bound function of the loss function L is derived as

$$
R_T = \sum_{t=1}^{T} L_t(w_t) - L_t(w^*) \le \sum_{t=1}^{T} \langle g_t, (w_t - w^*) \rangle
$$

where w^* is an ideal optimal weight and w_t is the weight found by HyAdamC at step t [4]. Moreover, $\langle a, b \rangle$ denotes an inner-product for a and b. Then, the upper bound of the inner product $\langle g_t, (w_t - w^*) \rangle$ is computed by

$$
\begin{aligned}
\langle g_t, (w_t - w^*) \rangle \leq\ & \frac{1}{2\alpha_t\eta_t(1-\beta_{1,t})}\left[\| \hat{V}_t^{1/4}(w_t - w^*) \|^2 - \| \hat{V}_t^{1/4}(w_{t+1} - w^*) \|^2\right] \\
&+ \frac{\alpha_t\eta_t}{2(1-\beta_{1,t})} \| \hat{V}_t^{-1/4} m_t \|^2 - \frac{\beta_{1,t}\langle m_{t-1}, w_t - w^* \rangle}{1-\beta_{1,t}} \\
\leq\ & \frac{1}{2\alpha_t\eta_t(1-\beta_{1,t})}\left[\| \hat{V}_t^{1/4}(w_t - w^*) \|^2 - \| \hat{V}_t^{1/4}(w_{t+1} - w^*) \|^2\right] \\
&+ \frac{\alpha_t\eta_t}{2(1-\beta_{1,t})} \| \hat{V}_t^{-1/4} m_t \|^2 + \frac{\beta_{1,t}\langle m_{t-1}, w_t - w^* \rangle}{1-\beta_{1,t}}
\end{aligned}
$$

where the final term in the second inequality satisfies

$$
\frac{\beta_{1,t}\langle m_{t-1}, w_t - w^* \rangle}{1-\beta_{1,t}} \leq \frac{\beta_{1,t}\alpha_t\eta_t \| \hat{V}_t^{-1/4} m_{t-1} \|^2}{2(1-\beta_{1,t})} + \frac{\beta_{1,t} \| \hat{V}_t^{1/4}(w_t - w^*) \|^2}{2\alpha_t\eta_t(1-\beta_{1,t})}.
$$

Therefore, we can further expand the upper bound of $\langle g_t, (w_t - w^*) \rangle$ as

$$
\begin{aligned}
\langle g_t, (w_t - w^*) \rangle \leq\ & \frac{1}{2\alpha_t\eta_t(1-\beta_{1,t})}\left[\| \hat{V}_t^{1/4}(w_t - w^*) \|^2 - \| \hat{V}_t^{1/4}(w_{t+1} - w^*) \|^2\right] \\
&+ \frac{\alpha_t\eta_t}{2(1-\beta_{1,t})} \| \hat{V}_t^{-1/4} m_t \|^2 \\
&+ \frac{\beta_{1,t}\alpha_t\eta_t \| \hat{V}_t^{-1/4} m_{t-1} \|^2}{2(1-\beta_{1,t})} + \frac{\beta_{1,t} \| \hat{V}_t^{1/4}(w_t - w^*) \|^2}{2\alpha_t\eta_t(1-\beta_{1,t})} \\
\leq\ & \frac{1}{2\alpha_t\eta_t(1-\beta_{1,t})}\left[\| \hat{V}_t^{1/4}(w_t - w^*) \|^2 - \| \hat{V}_t^{1/4}(w_{t+1} - w^*) \|^2\right] \\
&+ \frac{\alpha_t\eta_t}{2(1-\beta_{1,t})} \| \hat{V}_t^{-1/4} m_t \|^2 \\
&+ \frac{\alpha_t\eta_t}{2(1-\beta_{1,t})} \| \hat{V}_t^{-1/4} m_t \|^2 + \frac{\beta_{1,t} \| \hat{V}_t^{1/4}(w_t - w^*) \|^2}{2\alpha_t\eta_t(1-\beta_{1,t})} \\
\leq\ & \frac{1}{2\alpha_t\eta_t(1-\beta_{1,t})}\left[\| \hat{V}_t^{1/4}(w_t - w^*) \|^2 - \| \hat{V}_t^{1/4}(w_{t+1} - w^*) \|^2\right] \\
&+ \frac{\alpha_t\eta_t}{(1-\beta_{1,t})} \| \hat{V}_t^{-1/4} m_t \|^2 + \frac{\beta_{1,t} \| \hat{V}_t^{1/4}(w_t - w^*) \|^2}{2\alpha_t\eta_t(1-\beta_{1,t})}.
\end{aligned}
$$

Accordingly, the upper bound of R_t is given by

$$
\begin{aligned}
R_T \leq\ & \sum_{t=1}^{T} \langle g_t, (w_t - w^*) \rangle \\
\leq\ & \sum_{t=1}^{T} \frac{\| \hat{V}_t^{1/4}(w_t - w^*) \|^2 - \| \hat{V}_t^{1/4}(w_{t+1} - w^*) \|^2}{2\alpha_t\eta_t(1-\beta_{1,t})} \\
&+ \sum_{t=1}^{T} \frac{\alpha_t\eta_t \| \hat{V}_t^{-1/4} m_t \|^2}{(1-\beta_{1,t})} + \sum_{t=1}^{T} \frac{\beta_{1,t} \| \hat{V}_t^{1/4}(w_t - w^*) \|^2}{2\alpha_t\eta_t(1-\beta_{1,t})}.
\end{aligned}
$$

However, this upper bound has to be modified because the coefficient $\beta_{1,t}$ in HyAdamC does not satisfy $\forall_t, \beta_{1,t} \leq \beta_{1,t+1}$. That is, HyAdamC computes $\beta_{1,t}$ adaptively whenever each step is progressed. To address such adaptive coefficient problem in the proofs of [4], the authors of [3] applied a new term for the first momentum into the inequality of R_T. Accordingly, the Lemma 2 of [4] is refined as

$$\sum_{t=1}^{T} \alpha_t \eta_t \parallel \hat{V}_t^{-1/4} m_t \parallel^2 = \sum_{t=1}^{T-1} \alpha_t \eta_t \parallel \hat{V}_t^{-1/4} m_t \parallel^2 + \alpha_T \sum_{i=1}^{d} \frac{\eta_{T,i} m_{T,i}^2}{\sqrt{\hat{v}_{T,i}}}.$$

In this equation, the final term $\alpha_T \sum_{i=1}^{d} \frac{\eta_{T,i} m_{T,i}^2}{\sqrt{\hat{v}_{T,i}}}$ satisfies

$$\alpha_T \sum_{i=1}^{d} \frac{\eta_{T,i} m_{T,i}^2}{\sqrt{\hat{v}_{T,i}}} \leq \alpha \sum_{i=1}^{d} S_i.$$

Then, the upper bound of S_i is derived as

$$S_i \leq \frac{\eta_{T,i} \left[\sum_{j=1}^{T} \prod_{k=1}^{T-j} (1 - \beta_{1,T-k+1}) \beta_{1,T-k+1} g_{j,i} \right]^2}{\sqrt{T(1-\beta_2) \sum_{a=1}^{T} \beta_2^{T-a} \beta_{1,a-1}^2 (m_{a-1,i} - g_{a,i})^2}}$$

where $m_{a-1,i}$ is computed by

$$m_{a-1,i} = \sum_{j=1}^{a-1} \prod_{k=1}^{a-1-j} (1 - \beta_{1,a-k}) \beta_{1,a-k} g_{j,i}.$$

In this equation, the coefficient $(1 - \beta_{1,a-k})\beta_{1,a-k}$ always has a value between 0 and 0.25. Thus, $m_{a-1,i}$ is approximated as

$$\begin{aligned} m_{a-1,i} &= \sum_{j=1}^{a-1} \left(\prod_{k=1}^{a-1-j} (1 - \beta_{1,a-k}) \beta_{1,a-k} \right) g_{j,i} \\ &\lesssim \sum_{j=1}^{a-1} \left(\prod_{k=1}^{a-1-j} \frac{1}{4} \right) g_{j,i} \\ &= \sum_{j=1}^{a-1} \left(\frac{1}{4} \right)^{a-1-j} g_{j,i} \\ &\approx \sum_{j=1}^{a-1} \left(\frac{1}{4} \right)^{a-2} g_{j,i}. \end{aligned}$$

Then, we can express the inequality for S_i further concisely using the approximated $m_{a-1,i}$ as

$$S_i \lesssim \frac{\eta_{T,i}\left[\sum_{j=1}^{T}\prod_{k=1}^{T-j}(1-\beta_{1,T-k+1})\beta_{1,T-k+1}g_{j,i}\right]^2}{\sqrt{T(1-\beta_2)\sum_{a=1}^{T}\beta_2^{T-a}\beta_{1,a-1}^2(0.25^T\sum_{j=1}^{a-1}g_{j,i}-g_{a,i})^2}}$$
$$\approx \frac{\eta_{T,i}\left[\sum_{j=1}^{T}\prod_{k=1}^{T-j}(1-\beta_{1,T-k+1})\beta_{1,T-k+1}g_{j,i}\right]^2}{\sqrt{T(1-\beta_2)\sum_{a=1}^{T}\beta_2^{T-a}\beta_{1,a-1}^2 g_{a,i}^2}}.$$

In this equation, we can ignore 0.25^T because T is any sufficiently large value and 0.25^T is a very small value close to 0. Thus, the term $0.25^T\sum_{j=1}^{a-1}g_{j,i}$ is removed from the denominator. Accordingly, we can derive the upper bound of S_i further concretely by modifying its nominator, which is derived as

$$S_i \leq \frac{\eta_{T,i}\left[\sum_{j=1}^{T}\prod_{k=1}^{T-j}(1-\beta_{1,T-k+1})\beta_{1,T-k+1}g_{j,i}\right]^2}{\sqrt{T(1-\beta_2)\sum_{a=1}^{T}\beta_2^{T-a}\beta_{1,a-1}^2 g_{a,i}^2}}$$
$$\leq \frac{\eta_{T,i}\left(\sum_{j=1}^{T}\prod_{k=1}^{T-j}\beta_{1,T-k+1}\right)\left(\sum_{j=1}^{T}\prod_{k=1}^{T-j}\beta_{1,T-k+1}g_{j,i}^2\right)}{\sqrt{T(1-\beta_2)\sum_{a=1}^{T}\beta_2^{T-a}\beta_{1,a-1}^2 g_{a,i}^2}}$$
$$\leq \frac{\eta_{T,i}\left(\sum_{j=1}^{T}\bar{\beta}_w^{T-j}\right)\left(\sum_{j=1}^{T}\bar{\beta}_w^{T-j}g_{j,i}^2\right)}{\sqrt{T(1-\beta_2)\sum_{a=1}^{T}\beta_2^{T-a}\beta_{1,a-1}^2 g_{a,i}^2}}$$
$$\leq \frac{\eta_{T,i}\left(\sum_{j=1}^{T}\bar{\beta}_w^{T-j}\right)\left(\sum_{j=1}^{T}\bar{\beta}_w^{T-j}g_{j,i}^2\right)}{\sqrt{T(1-\beta_2)\beta_{min}^2\sum_{a=1}^{T}\beta_2^{T-a}g_{a,i}^2}}$$

where $\beta_{min} = min\{\beta_{1,1}, \beta_{1,2}, ..., \beta_{1,T-1}\}$. Hence, we get

$$
\begin{aligned}
&\alpha_T \sum_{i=1}^{d} \frac{\eta_{T,i} m_{T,i}^2}{\sqrt{\hat{v}_{T,i}}} \\
&\quad \leq \alpha \sum_{i=1}^{d} S_i \\
&\quad \leq \alpha \sum_{i=1}^{d} \frac{\eta_{T,i} \left(\sum_{j=1}^{T} \bar{\beta}_w^{T-j}\right)\left(\sum_{j=1}^{T} \bar{\beta}_w^{T-j} g_{j,i}^2\right)}{\sqrt{T(1-\beta_2)\beta_{min}^2 \sum_{j=1}^{T} \beta_2^{T-j} g_{j,i}^2}} \\
&\quad \leq \frac{\alpha(1-\bar{\beta}_w^T)}{(1-\bar{\beta}_w)|\beta_{min}|\sqrt{T(1-\beta_2)}} \sum_{i=1}^{d}\sum_{j=1}^{T} \frac{\eta_{T,i}\bar{\beta}_w^{T-j} g_{j,i}^2}{\sqrt{\beta_2^{T-j} g_{j,i}^2}} \\
&\quad \leq \frac{\alpha(1-\bar{\beta}_w^T)}{(1-\bar{\beta}_w)|\beta_{min}|\sqrt{T(1-\beta_2)}} \sum_{i=1}^{d}\sum_{j=1}^{T} \eta_{T,i} \left(\frac{\bar{\beta}_w}{\sqrt{\beta_2}}\right)^{T-j} |g_{j,i}| \\
&\quad \leq \frac{\alpha}{(1-\bar{\beta}_w)|\beta_{min}|\sqrt{T(1-\beta_2)}} \sum_{i=1}^{d}\sum_{j=1}^{T} \eta_{T,i}\gamma^{T-j}|g_{j,i}|
\end{aligned}
$$

where $\gamma = \bar{\beta}_w/\sqrt{\beta_2}$.

Using the above inequality, we modify the Lemma 2 of [4] so as to satisfy the condition of HyAdamC as

$$
\begin{aligned}
&\sum_{t=1}^{T} \alpha_t \eta_t \parallel \hat{V}_t^{-1/4} m_t \parallel^2 \\
&\quad = \sum_{t=1}^{T-1} \alpha_t \eta_t \parallel \hat{V}_t^{-1/4} m_t \parallel^2 + \alpha_T \sum_{i=1}^{d} \frac{\eta_{T,i} m_{T,i}^2}{\sqrt{\hat{v}_{T,i}}} \\
&\quad \leq \alpha \sum_{t=1}^{T} \frac{\sum_{i=1}^{d}\sum_{j=1}^{t} \eta_{T,i}\gamma^{t-j}|g_{j,i}|}{(1-\bar{\beta}_w)|\beta_{min}|\sqrt{t(1-\beta_2)}} \\
&\quad \leq \frac{\alpha\sqrt{1+\log T}}{(1-\bar{\beta}_w)|\beta_{min}|(1-\gamma)\sqrt{(1-\beta_2)}} \sum_{i=1}^{d} \eta_{T,i} \left\|g_{1:T,i}\right\|_2 \\
&\quad \leq \frac{\alpha\sqrt{1+\log T}}{2(1-\bar{\beta}_w)|\beta_{min}|(1-\gamma)\sqrt{(1-\beta_2)}} \sum_{i=1}^{d} \left\|g_{1:T,i}\right\|_2 .
\end{aligned}
$$

In this inequality, $\eta_{T,i}$ can be replaced by 0.5 because T is a sufficiently large value. In detail, the initial-term velocity control function $\zeta_{I,t}$ converges to a value close to 1 after the steps are sufficiently progressed. Then, $\zeta_{I,T} \approx 1$. Likewise, in the short-term velocity control function $\zeta_{S,t}$, $|\mathbf{g}_T - \mathbf{g}_{T-1}|$ converges to a value close to 0 because $\mathbf{g}_t$ becomes almost equivalent to $\mathbf{g}_{t-1}$ after the step is sufficiently progressed. In other words, $\eta_{T,i} \approx 0.5$ due to $\zeta_{S,T} \approx 0.5$.

Now, we rewrite the upper bound of R_T by applying the above modified Lemma 2 of [4] as

$$
\begin{aligned}
R_T \le& \sum_{t=1}^{T} \frac{\parallel \hat{V}_t^{1/4}(w_t - w^*) \parallel^2 - \parallel \hat{V}_t^{1/4}(w_{t+1} - w^*) \parallel^2}{2\alpha_t \eta_t (1-\beta_{1,t})} \\
&+ \sum_{t=1}^{T} \frac{\beta_{1,t} \parallel \hat{V}_t^{1/4}(w_t - w^*) \parallel^2}{2\alpha_t \eta_t (1-\beta_{1,t})} + \sum_{t=1}^{T} \frac{\alpha_t \eta_t \parallel \hat{V}_t^{-1/4} m_t \parallel^2}{1-\beta_{1,t}} \\
\le& \sum_{t=1}^{T} \frac{\parallel \hat{V}_t^{1/4}(w_t - w^*) \parallel^2 - \parallel \hat{V}_t^{1/4}(w_{t+1} - w^*) \parallel^2}{2\alpha_t \eta_t (1-\beta_{1,t})} \\
&+ \sum_{t=1}^{T} \frac{\beta_{1,t} \parallel \hat{V}_t^{1/4}(w_t - w^*) \parallel^2}{2\alpha_t \eta_t (1-\beta_{1,t})} + \sum_{t=1}^{T} \frac{\alpha_t \eta_t \parallel \hat{V}_t^{-1/4} m_t \parallel^2}{1-\bar{\beta}_w} \\
\le& \sum_{t=1}^{T} \frac{\parallel \hat{V}_t^{1/4}(w_t - w^*) \parallel^2 - \parallel \hat{V}_t^{1/4}(w_{t+1} - w^*) \parallel^2}{2\alpha_t \eta_t (1-\beta_{1,t})} \\
&+ \sum_{t=1}^{T} \frac{\beta_{1,t} \parallel \hat{V}_t^{1/4}(w_t - w^*) \parallel^2}{2\alpha_t \eta_t (1-\beta_{1,t})} \\
&+ \frac{\alpha\sqrt{1+\log T}}{2(1-\bar{\beta}_w)^2 |\beta_{min}| (1-\gamma)\sqrt{(1-\beta_2)}} \sum_{i=1}^{d} \left\| g_{1:T,i} \right\|_2 \\
\le& \sum_{t=1}^{T} \frac{\parallel \hat{V}_t^{1/4}(w_t - w^*) \parallel^2 - \parallel \hat{V}_t^{1/4}(w_{t+1} - w^*) \parallel^2}{2\alpha_t \eta_t (1-\beta_{1,t})} \\
&+ \sum_{t=1}^{T} \frac{\beta_{1,t} \parallel \hat{V}_t^{1/4}(w_t - w^*) \parallel^2}{2\alpha_t \eta_t (1-\beta_{1,t})} \\
&+ \frac{\alpha\sqrt{1+\log T}}{2(1-\bar{\beta}_w)^2 |\beta_{min}| (1-\gamma)\sqrt{(1-\beta_2)}} \sum_{i=1}^{d} \left\| g_{1:T,i} \right\|_2 .
\end{aligned}
$$

In this inequality, let

$$
\begin{aligned}
R_{A,t} &= \frac{\parallel \hat{V}_t^{1/4}(w_t - w^*) \parallel^2 - \parallel \hat{V}_t^{1/4}(w_{t+1} - w^*) \parallel^2}{2\alpha_t \eta_t (1-\beta_{1,t})} \\
R_{B,t} &= \frac{\beta_{1,t} \parallel \hat{V}_t^{1/4}(w_t - w^*) \parallel^2}{2\alpha_t \eta_t (1-\beta_{1,t})} \\
R_C &= \frac{\alpha\sqrt{1+\log T}}{2(1-\bar{\beta}_w)^2 |\beta_{min}| (1-\gamma)\sqrt{(1-\beta_2)}} \sum_{i=1}^{d} \left\| g_{1:T,i} \right\|_2 .
\end{aligned}
$$

Then, R_T is concisely expressed as

$$R_T \leq \sum_{t=1}^{T} R_{A,t} + \sum_{t=1}^{T} R_{B,t} + R_C.$$

Henceforth, we further expand the upper bound of R_T by analyzing the bounds of $\sum_{t=1}^{T} R_{A,t}$ and $\sum_{t=1}^{T} R_{B,t}$. First, we derive the upper bound of $\sum_{t=1}^{T} R_{A,t}$ as

$$\begin{aligned}
\sum_{t=1}^{T} R_{A,t} &= \sum_{t=1}^{T} \frac{\| \hat{V}_t^{1/4}(w_t - w^*) \|^2 - \| \hat{V}_t^{1/4}(w_{t+1} - w^*) \|^2}{2\alpha_t \eta_t (1-\beta_{1,t})} \\
&\leq \sum_{t=1}^{T} \frac{\| \hat{V}_t^{1/4}(w_t - w^*) \|^2 - \| \hat{V}_t^{1/4}(w_{t+1} - w^*) \|^2}{2\alpha_t \eta_t (1-\bar{\beta}_w)} \\
&= \frac{1}{2(1-\bar{\beta}_w)} \sum_{t=1}^{T} \frac{\| \hat{V}_t^{1/4}(w_t - w^*) \|^2 - \| \hat{V}_t^{1/4}(w_{t+1} - w^*) \|^2}{\alpha_t \eta_t} \\
&\leq \frac{1}{2(1-\bar{\beta}_w)} \sum_{t=1}^{T} \left[\frac{\| \hat{V}_t^{1/4}(w_t - w^*) \|^2}{\alpha_t \eta_t} - \frac{\| \hat{V}_t^{1/4}(w_{t+1} - w^*) \|^2}{\alpha_t \eta_t} \right] \\
&\leq \frac{\| \hat{V}_1^{1/4}(w_1 - w^*) \|^2}{2\alpha_1 \eta_1 (1-\bar{\beta}_w)} \\
&\quad + \frac{1}{2(1-\bar{\beta}_w)} \sum_{t=2}^{T} \left[\frac{\| \hat{V}_t^{1/4}(w_t - w^*) \|^2}{\alpha_t \eta_t} - \frac{\| \hat{V}_{t-1}^{1/4}(w_t - w^*) \|^2}{\alpha_{t-1} \eta_{t-1}} \right] \\
&\leq \frac{1}{2\alpha_1(1-\bar{\beta}_w)} \sum_{i=1}^{d} \frac{\sqrt{\hat{v}_{1,i}}(w_{1,i} - w_i^*)}{\eta_{1,i}} \\
&\quad + \frac{\sum_{t=2}^{T} \sum_{i=1}^{d} (w_{t,i} - w_i^*)^2 \left(\sqrt{\hat{v}_{t,i}} \alpha_t^{-1} \eta_t^{-1} - \sqrt{\hat{v}_{t-1,i}} \alpha_{t-1}^{-1} \eta_{t-1}^{-1} \right)}{2(1-\bar{\beta}_w)} \\
&\leq \frac{D_\infty^2}{2\alpha_1(1-\bar{\beta}_w)} \sum_{i=1}^{d} \frac{\sqrt{\hat{v}_{1,i}}}{\eta_{1,i}} \\
&\quad + \frac{D_\infty^2}{2(1-\bar{\beta}_w)} \sum_{t=2}^{T} \sum_{i=1}^{d} \left(\sqrt{\hat{v}_{t,i}} \alpha_t^{-1} \eta_{t,i}^{-1} - \sqrt{\hat{v}_{t-1,i}} \alpha_{t-1}^{-1} \eta_{t-1,i}^{-1} \right).
\end{aligned}$$

This inequality is further simplified by the telescopic sum as

$$\begin{aligned}
\sum_{t=1}^{T} R_{A,t} &\leq \frac{D_\infty^2}{2\alpha_T(1-\bar{\beta}_w)} \sum_{i=1}^{d} \frac{\sqrt{\hat{v}_{T,i}}}{\eta_{T,i}} \\
&\approx \frac{D_\infty^2}{4\alpha_T(1-\bar{\beta}_w)} \sum_{i=1}^{d} \sqrt{\hat{v}_{T,i}}.
\end{aligned}$$

Second, we derive the upper bound of $\sum_{t=1}^{T} R_{B,t}$ as

$$
\begin{aligned}
\sum_{t=1}^{T} R_{B,t} &= \sum_{t=1}^{T} \frac{\beta_{1,t} \parallel \hat{V}_t^{1/4}(w_t - w^*) \parallel^2}{2\alpha_t \eta_t (1-\beta_{1,t})} \\
&\leq \frac{1}{2(1-\bar{\beta}_w)} \sum_{t=1}^{T} \frac{\beta_{1,t} \parallel \hat{V}_t^{1/4}(w_t - w^*) \parallel^2}{\alpha_t \eta_t} \\
&\leq \sum_{t=1}^{T} \frac{\beta_{1,t} \parallel \hat{V}_t^{1/4}(w_t - w^*) \parallel^2}{\alpha_t \eta_t (1-\bar{\beta}_w)^2} \\
&\leq \frac{1}{(1-\bar{\beta}_w)^2} \sum_{t=1}^{T} \sum_{i=1}^{d} \beta_{1,t} (w_t - w^*) \sqrt{\hat{v}_{t,i}} \alpha_t^{-1} \eta_{t,i}^{-1} \\
&\leq \frac{D_\infty^2}{(1-\bar{\beta}_w)^2} \sum_{t=1}^{T} \sum_{i=1}^{d} \beta_{1,t} \sqrt{\hat{v}_{t,i}} \alpha_t^{-1} \eta_{t,i}^{-1}.
\end{aligned}
$$

Third, in order to derive the upper bound of R_T, we combine the bounds of $\sum_{t=1}^{T} R_{A,t}$, $\sum_{t=1}^{T} R_{B,t}$, and $R_{C,T}$ into one as

$$
\begin{aligned}
R_T &\leq \sum_{t=1}^{T} R_{A,t} + \sum_{t=1}^{T} R_{B,t} + R_C \\
&\leq \frac{D_\infty^2}{4\alpha_T(1-\bar{\beta}_w)} \sum_{i=1}^{d} \sqrt{\hat{v}_{T,i}} + \frac{D_\infty^2}{(1-\bar{\beta}_w)^2} \sum_{t=1}^{T} \sum_{i=1}^{d} \frac{\beta_{1,t}\sqrt{\hat{v}_{t,i}}}{\alpha_t \eta_{t,i}} \\
&\quad + \frac{\alpha\sqrt{1+\log T}}{2(1-\bar{\beta}_w)^2 |\beta_{min}| (1-\gamma)\sqrt{(1-\beta_2)}} \sum_{i=1}^{d} \left\| g_{1:T,i} \right\|_2 \\
&\leq \frac{D_\infty^2}{\alpha_T(1-\bar{\beta}_w)} \sum_{i=1}^{d} \sqrt{\hat{v}_{T,i}} + \frac{D_\infty^2}{(1-\bar{\beta}_w)^2} \sum_{t=1}^{T} \sum_{i=1}^{d} \frac{\beta_{1,t}\sqrt{\hat{v}_{t,i}}}{\alpha_t \eta_{t,i}} \\
&\quad + \frac{\alpha\sqrt{1+\log T}}{(1-\bar{\beta}_w)^2 |\beta_{min}| (1-\gamma)\sqrt{(1-\beta_2)}} \sum_{i=1}^{d} \left\| g_{1:T,i} \right\|_2 \\
&= \frac{D_\infty^2}{\alpha_T(1-\bar{\beta}_w)} \sum_{i=1}^{d} \sqrt{\hat{v}_{T,i}} + \frac{D_\infty^2}{(1-\bar{\beta}_w)^2} \sum_{t=1}^{T} \sum_{i=1}^{d} \frac{\beta_{1,t}\eta_{S,t,i}^{-1}\sqrt{\hat{v}_{t,i}}}{\alpha_t \eta_{I,t}} \\
&\quad + \frac{\alpha\sqrt{1+\log T}}{(1-\bar{\beta}_w)^2 |\beta_{min}| (1-\gamma)\sqrt{(1-\beta_2)}} \sum_{i=1}^{d} \left\| g_{1:T,i} \right\|_2
\end{aligned}
$$

where $\eta_{t,i} = \eta_{S,t}\eta_{S,t,i}$ and $\eta_{S,t}$ is independent to the index $i \in \{1, ..., d\}$.

Hence, we have proved that the upper regret bound of HyAdamC is

$$R_T \leq \frac{D_\infty^2}{\alpha_T(1-\bar{\beta}_w)} \sum_{i=1}^{d} \sqrt{\hat{v}_{T,i}} + \frac{D_\infty^2}{(1-\bar{\beta}_w)^2} \sum_{t=1}^{T} \sum_{i=1}^{d} \frac{\beta_{1,t}\eta_{S,t,i}^{-1}\sqrt{\hat{v}_{t,i}}}{\alpha_t \eta_{I,t}}$$

$$+ \frac{\alpha\sqrt{1+\log T}}{(1-\bar{\beta}_w)^2 |\beta_{min}| (1-\gamma)\sqrt{(1-\beta_2)}} \sum_{i=1}^{d} \left\| g_{1:T,i} \right\|_2 .$$

References

[1] William R Bell and Elizabeth T Huang. Using the t-distribution to deal with outliers in small area estimation. In *Proceedings of Statistics Canada Symposium*, 2006.

[2] Robert V Hogg, Joseph McKean, and Allen T Craig. *Introduction to mathematical statistics*. Pearson Education, 2005.

[3] Wendyam Eric Lionel Ilboudo, Taisuke Kobayashi, and Kenji Sugimoto. Robust stochastic gradient descent with student-t distribution based first-order momentum. *IEEE Transactions on Neural Networks and Learning Systems*, pages 1–14, 2020.

[4] Sashank J. Reddi, Satyen Kale, and Sanjiv Kumar. On the convergence of adam and beyond, 2019.

[5] Wonkeun Youn, Yulong Huang, and Hyun Myung. Outlier-robust student's-t-based imm-vb localization for manned aircraft using tdoa measurements. *IEEE/ASME Transactions on Mechatronics*, 25(3):1646–1658, 2020.

[6] Martin Zinkevich. Online convex programming and generalized infinitesimal gradient ascent. In *Proceedings of the 20th international conference on machine learning (icml-03)*, pages 928–936, 2003.
